# Daily oral administration of probiotics engineered to constantly secrete short-chain fatty acids effectively prevents myocardial injury from subsequent ischaemic heart disease

**DOI:** 10.1093/cvr/cvae128

**Published:** 2024-06-08

**Authors:** Quynh Hoa Pham, Thi Van Anh Bui, Woo-Sup Sim, King Hoo Lim, Carmen Oi Kwan Law, Wanyu Tan, Ri Youn Kim, Kwan Ting Chow, Hun-Jun Park, Kiwon Ban, Terrence Chi Kong Lau

**Affiliations:** Department of Biomedical Sciences, College of Veterinary Medicine and Life Science, City University of Hong Kong, 83 Tat Chee Avenue, Hong Kong Special Administrative Region; Tung Biomedical Sciences Centre, City University of Hong Kong, 83 Tat Chee Avenue, Hong Kong Special Administrative Region; Department of Biomedical Sciences, College of Veterinary Medicine and Life Science, City University of Hong Kong, 83 Tat Chee Avenue, Hong Kong Special Administrative Region; Tung Biomedical Sciences Centre, City University of Hong Kong, 83 Tat Chee Avenue, Hong Kong Special Administrative Region; Department of Biomedicine and Health Sciences, The Catholic University of Korea, 222 Banpo-daero, Seocho-gu, Seoul 137701, Korea; Division of Cardiology, Department of Internal Medicine, Seoul St. Mary’s Hospital, The Catholic University of Korea, 222 Banpo-daero, Seocho-gu, Seoul 137701, Korea; Department of Biomedical Sciences, College of Veterinary Medicine and Life Science, City University of Hong Kong, 83 Tat Chee Avenue, Hong Kong Special Administrative Region; Tung Biomedical Sciences Centre, City University of Hong Kong, 83 Tat Chee Avenue, Hong Kong Special Administrative Region; Department of Biomedical Sciences, College of Veterinary Medicine and Life Science, City University of Hong Kong, 83 Tat Chee Avenue, Hong Kong Special Administrative Region; Tung Biomedical Sciences Centre, City University of Hong Kong, 83 Tat Chee Avenue, Hong Kong Special Administrative Region; Department of Biomedical Sciences, College of Veterinary Medicine and Life Science, City University of Hong Kong, 83 Tat Chee Avenue, Hong Kong Special Administrative Region; Tung Biomedical Sciences Centre, City University of Hong Kong, 83 Tat Chee Avenue, Hong Kong Special Administrative Region; Department of Biomedical Sciences, College of Veterinary Medicine and Life Science, City University of Hong Kong, 83 Tat Chee Avenue, Hong Kong Special Administrative Region; Tung Biomedical Sciences Centre, City University of Hong Kong, 83 Tat Chee Avenue, Hong Kong Special Administrative Region; Department of Biomedical Sciences, College of Veterinary Medicine and Life Science, City University of Hong Kong, 83 Tat Chee Avenue, Hong Kong Special Administrative Region; Tung Biomedical Sciences Centre, City University of Hong Kong, 83 Tat Chee Avenue, Hong Kong Special Administrative Region; Department of Biomedicine and Health Sciences, The Catholic University of Korea, 222 Banpo-daero, Seocho-gu, Seoul 137701, Korea; Division of Cardiology, Department of Internal Medicine, Seoul St. Mary’s Hospital, The Catholic University of Korea, 222 Banpo-daero, Seocho-gu, Seoul 137701, Korea; Department of Biomedical Sciences, College of Veterinary Medicine and Life Science, City University of Hong Kong, 83 Tat Chee Avenue, Hong Kong Special Administrative Region; Tung Biomedical Sciences Centre, City University of Hong Kong, 83 Tat Chee Avenue, Hong Kong Special Administrative Region; Department of Biomedical Sciences, College of Veterinary Medicine and Life Science, City University of Hong Kong, 83 Tat Chee Avenue, Hong Kong Special Administrative Region; Tung Biomedical Sciences Centre, City University of Hong Kong, 83 Tat Chee Avenue, Hong Kong Special Administrative Region

**Keywords:** Coronary heart disease, Myocardial infarction, Prevention, Probiotics, Short-chain fatty acid

## Abstract

**Aims:**

Given the extremely limited regeneration potential of the heart, one of the most effective strategies to reduce the prevalence and mortality of coronary artery disease is prevention. Short-chain fatty acids (SCFAs), which are by-products of beneficial probiotics, have been reported to possess cardioprotective effects. Despite their beneficial roles, delivering SCFAs and maintaining their effective concentration in plasma present major challenges. Therefore, in the present study, we aimed to devise a strategy to prevent coronary heart disease effectively by using engineered probiotics to continuously release SCFAs *in vivo*.

**Methods and results:**

We engineered a novel probiotic cocktail, namely EcN_TL, from the commercially available *Escherichia coli* Nissle 1917 (EcN) strain to continuously secrete SCFAs by introducing the propionate and butyrate biosynthetic pathways. Oral administration of EcN_TL enhanced and maintained an effective concentration of SCFAs in the plasma. As a preventative strategy, we observed that daily intake of EcN_TL for 14 days prior to ischaemia–reperfusion injury significantly reduced myocardial injury and improved cardiac performance compared with EcN administration. We uncovered that EcN_TL’s protective mechanisms included reducing neutrophil infiltration into the infarct site and promoting the polarization of wound healing macrophages. We further revealed that SCFAs at plasma concentration protected cardiomyocytes from inflammation by suppressing the NF-κB activation pathway.

**Conclusion:**

These data provide strong evidence to support the use of SCFA-secreting probiotics to prevent coronary heart disease. Since SCFAs also play a key role in other metabolic diseases, EcN_TL can potentially be used to treat a variety of other diseases.

## Graphical Abstract

**Graphical Abstract:**
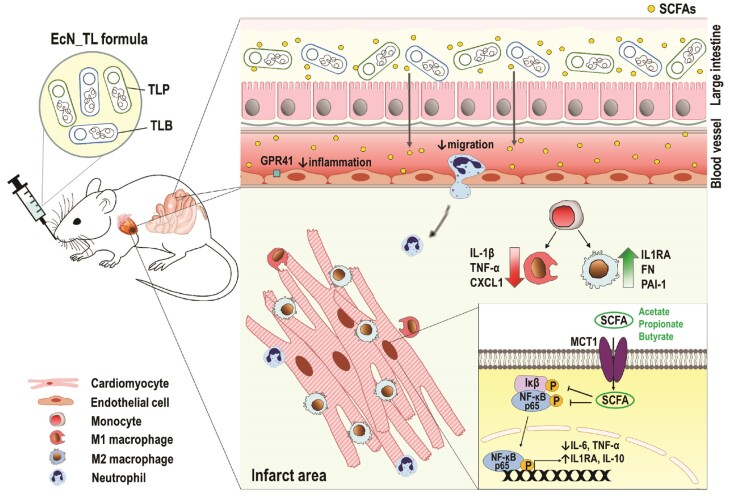
Schematic diagram showing the therapeutic effect of engineer probiotic EcN_TL for myocardial infarction.

**Time of primary review: 52 days**

## 1. Introduction

Coronary heart disease (CHD), including myocardial infarction (MI), is a leading cause of global mortality, accounting for more than 800 000 deaths per year.^1^ The irreversible damage of MI to the heart diminishes cardiac contractile function and leads to a 5-year survival rate of only 49% from the onset of symptoms.^2^ Due to the extremely limited regeneration potential of the heart, current therapies, including surgical and pharmacological interventions, can only delay the progression of this detrimental disease.^2^ Therefore, prevention is the best strategy for reducing the prevalence and mortality of CHD. In light of this, various potential therapeutic approaches have been developed and explored to effectively prevent CHD. However, apart from lifestyle changes, none of these strategies have consistently yielded significant and reliable outcomes.

Several lines of evidence indicated that the gut microbiome plays a crucial role in regulating health and disease conditions by modulating homoeostasis or producing beneficial compounds, such as short-chain fatty acids (SCFAs).^3,4^ SCFAs, which are fatty acids with fewer than six carbons in length, serve as the major nutrient source for the caeco-colonic epithelium and are produced by the gut microbiota within the distal intestinal tract. The three most prevalent SCFAs, namely, acetic acid, propionic acid, and butyric acid, are important end products of colonic fermentation. They are derived from macronutrients such as dietary fibre, resistant starches, and sugars or proteins that escape digestion in the upper intestinal tract. Recently, SCFAs have been shown to influence cardiac health by inhibiting inflammation, balancing gene regulation, and modulation of immune cell activation.^3,4^ In a mouse model of hypertension, both high-fibre diet and acetate supplementation significantly reduced blood pressure, as well as cardiac and renal fibrosis.^5,6^ Moreover, treatment with propionate substantially attenuated hypertension, cardiac hypertrophy, fibrosis, and vascular dysfunction in two independent experimental models of hypertension and atherosclerosis.^7^ These results provide strong evidence that SCFAs protect against adverse cardiac hypertrophy and the progression of fibrosis.^8–15^

Despite the numerous beneficial effects of SCFAs on human health, maintaining a physiological concentration of SCFAs in the plasma has been a major challenge as they are rapidly metabolized and used as an energy source throughout the body. Indeed, previous approaches, including intravenous injection, oral administration of water-dissolved or microencapsulated SCFAs, or the use of pro-drugs, have failed to achieve satisfactory outcomes.^16–20^ While the addition of fermentable fibres or chemically modified resistant starch to the diet has demonstrated a reasonable delivery of SCFAs, variations among individuals have been observed due to the diverse gut microbiota.^21,22^ Therefore, the development of effective and targeted delivery systems for SCFAs is highly demanded.

In this study, we aimed to develop a novel system for the effective delivery of SCFAs *in vivo* by utilizing probiotics engineered to continuously release SCFAs. To achieve this, we obtained the commercially available *Escherichia coli* Nissle 1917 (EcN) probiotic strain, also known as Mutaflor, as the source material for engineering. Subsequently, we successfully created a novel probiotic cocktail named EcN_TL that efficiently colonized the gut and maintained a stable and effective SCFA concentration in the plasma in a mouse model. Daily administration of EcN_TL demonstrated a significant preventive effect against myocardial ischaemia–reperfusion (I/R) injury. Furthermore, we discovered that specific formulations of SCFAs directly protected cardiomyocytes against inflammation, revealing the potential mechanism of SCFAs in cardiac protection and the crosstalk between the gut microbiota and the heart.

## 2. Methods

### 2.1 Genetic manipulation and culture of bacteria

The plasmid pQH was generated by removing *lacI* gene and adding *hok/sok* system into plasmid pACYCT2, which served as the backbone. For the construction of pTLP, the sleeping beauty mutase (*sbm*) operon is amplified from *E. coli* K-12 via polymerase chain reaction (PCR) and cloned into pQH plasmid using the CloneExpress Ultra One Step Cloning Kit (Vazyme). Similarly, for the construction of pTLB, the genes *atoB*, *crt*-*bcd-etfA-etfB-BHBD*, and *ptb-buk* were amplified from *E. coli* K-12, *Clostridium butyricum*, and *Clostridium acetobutyricum*, respectively. The genes were then arranged in order on the pQH plasmid. Both pathways were placed downstream of a strong constitutive promoter, pbBBa_J23119.^23^  Supplementary material online, *Figures S15* and *S16* provide the maps of the two plasmids, pTLP and pTLB.

The gene deletion in EcN was performed using the lambda red system, as described previously.^24^ Briefly, the EcN containing plasmid pSIJ8-Cam^R^ was cultured at 30°C. Induction of the lambda red system was achieved by adding arabinose to a final concentration of 15 mM and incubating for 30 min. The PCR product of the kanamycin-resistant cassette, with 50 bp (40 bp with *pta* gene) homologue to the target genes, was then electroporated into EcN/pSIJ8-Cam^R^ and allowed to recover overnight. The recombinants were selected on agar plates containing kanamycin at a concentration of 50 µg/mL. Confirmation of target gene disruption was performed through PCR and sequencing of the target genes. The primer sequences used for plasmid construction and gene disruption are shown in Supplementary material online, *Table S1*.

To generate the constitutive luminescent EcN, a method similar to the one described by Riedel *et al*.^25^ was employed. This involved using the plasmid pQH-ml-dual, which carries the bacterial luminescence cassette of *Photorhabdus luminescens*.

### 2.2 Probiotic administration

The mice study was approved by the Institutional Animal Care and Use Committee (IACUC) of the Catholic University of Korea (approval number: CUMC-2021-0243-06). All animal procedures conformed to the National Institutes of Health (NIH) guidelines or the guidelines issued by Directive 2010/63/EU of the European Parliament for the protection of animals used in scientific research. Eight-week-old C57BL/6J mice were randomly assigned to different groups. The mice were pre-treated with an antibiotic cocktail in their drinking water as described previously.^12^ The antibiotic cocktail consisted of vancomycin 0.125 g/L, neomycin 0.25 g/L, ampicillin 0.25 g/L, metronidazole 0.25 g/L, and 1% sucrose and was administered in the drinking water for a duration of 1 week. Prior to oral gavage, the mice were anaesthetized with 2% inhaled isoflurane. A suspension of 10^9^ colony-forming units (cfu) of bacteria in 200 μL phosphate-buffered saline (PBS) was then administered through the oesophagus using an 18 G stainless steel feeding needle with a 2.25 mm ball (THOMAS, USA). The luminescent signal of the bacteria in the abdominal region was monitored daily using the spectrum *in vivo* imaging system (IVIS; PerkinElmer). Mice were observed for at least 15 min immediately after oral gavage. At the end of the experiment, mice were sacrificed by inhaling 5% isoflurane. Subsequently, blood samples were collected from the right ventricle, and the cecum was also collected. The blood samples were then analysed for SCFA levels using gas chromatography (GC)-mass spectrometry (MS).

### 2.3 SCFA analysis by GC-MS

The culture media were collected by centrifugation to remove bacteria cells and then snap-frozen in liquid nitrogen. Blood serum and caecal samples were collected immediately after sacrificing the animals and stored at −80°C until analysis. The caecal content was homogenized with 1 mL of water and centrifuged at 12 000 rpm for 10 min at 4°C. For GC-MS analysis, the published protocol was followed.^26^ Briefly, 20 μL of supernatant or serum was derivatized by pentafluorobenzyl bromide (Sigma) in acetone at 60°C for 30 min. The derivatized product was then extracted using a solvent extraction method with hexane and water. GC-MS analysis was performed using the Agilent 6890N GC coupled with 5975 inert Performance Turbo MSD (Agilent Technologies) in the selected ion monitoring mode. SCFAs were identified by comparing retention time of sample peaks with external standards such as acetic acid, propionic acid, and butyric acid (Sigma). An external standard calibration was established to calculate the concentration of the sample.

### 2.4 I/R injury model

The rat studies were approved by the IACUC of the Catholic University of Korea (approval number: CUMC-2020-0051-01). All animal procedures conformed to the NIH guidelines, or the guidelines issued by Directive 2010/63/EU of the European Parliament for the protection of animals used in scientific research. Fisher 344 rats (160–180 g, 8-week-old males, Koatec, Korea) were given an antibiotic cocktail comprising vancomycin (0.125 g/L), neomycin (0.25 g/L), ampicillin (0.25 g/L), and metronidazole (0.25 g/L) in their drinking water for a week, similar to the mouse experiment. Afterwards, the rats were administered 10^11^ cfu of probiotics in 1 mL PBS through the oesophagus using the 18 G stainless steel feeding needle. The probiotics were given to the rats daily for 7 days before and after the surgery. For performing I/R injury model, the rats were anaesthetized with 2% inhaled isoflurane and intubated with an 18 G intravenous catheter. The rats were ventilated with a rodent respirator (Harvard Apparatus), and a 37°C heating pad was used to maintain their body temperature throughout the operation. The chest was shaved and sterilized with 70% alcohol. I/R was induced temporarily by occluding the left anterior descending (LAD) artery with a 7-0 Prolene suture for 1 h. The chest was aseptically closed and disinfected following the surgery; to establish the baseline, left ventricular (LV) function, ejection fraction (EF), and fractional shortening (FS) were examined 4 h after surgery.

### 2.5 Immunofluorescence staining

The heart sections were incubated in blocking solution for 1 h at room temperature and then incubated at 4°C overnight with one of the following antibodies: goat anti-CD31(1:200; R&D Cat #AF3628), rabbit anti-myeloperoxidase (MPO) (1:50; Abcam Cat #ab9535), mouse anti-CD68 (1:100; Abcam Cat #ab955), mouse anti-iNOS (1:200; Abcam Cat #ab49999), rabbit anti-CD206 (1:100; Abcam Cat #ab64693), and mouse anti-cTnT (1:200; Abcam Cat #ab8295). Confocal images were captured at room temperature with ZEN software on an upright confocal microscope (LSM 700; Carl Zeiss) with the predefined ZEN software configurations for Alexa Fluor 546, Alexa Fluor 488, and DAPI.

### 2.6 TUNEL assay

A terminal deoxynucleotidyl transferase–mediated dUTP nick end-labelling (TUNEL) kit (Roche; Cat #11684795910) assay was used to identify apoptosis. The sections were deparaffinized with xylene and rehydration. Then, the sections were permeabilized with 200 μL of TBS-T for 2 min on ice. The sections were incubated in a staining solution containing deoxynucleotidyl transferase for 1 h at 37°C in the dark. After washing with PBS, the sections were mounted with DAPI mounting solution (Vector; H-1500)

### 2.7 Echocardiography

The animals were anaesthetized with 2% isoflurane and placed on a heating pad to maintain the body temperature at 37°C. Serial echocardiography was performed baseline (4 h) and at 1, 2, and 4 weeks after the treatment using a transthoracic echocardiography system equipped with a 15 MHz L15-7io linear transducer (Affiniti 50G, Philips) to determine the EF, FS, LV internal diameter at end-diastole, LV internal diameter at end-systole, septal wall thickness, and posterior wall thickness. The echocardiography operator was blinded to the group allocation during the experiment.


EF(%)=[(LVEDV−LVESV)/LVEDV]*100,

FS(%)=[(LVEDD−LVESD)/LVEDD]*100.

### 2.8 Haemodynamic measurements

Haemodynamic measurements were performed at the endpoint of 8 weeks before euthanasia. Rats were anaesthetized and ventilated as described previously. After thoracotomy without bleeding, the LV apex of the heart was punctured with a 26 G needle and the 2F conductance catheter (SPR-838, Millar) was inserted into the LV. LV pressure–volume (PV) parameters were continually recorded using a PV conductance system (MPVS Ultra, emka TECHNOLOGIES, Paris, France) coupled to a digital converter (PowerLab 16/35, ADInstruments, Colorado Springs, CO). Load-independent parameters of cardiac function including the slopes of end-systolic pressure–volume relationship (ESPVR) and end-diastolic pressure–volume relationship (EDPVR) were measured at different preloads, which were elicited by transient occlusion of the inferior vena cava with needle holder. Fifty microlitres of hypertonic saline (20% 22 NaCl) was injected into the left jugular vein to calculate the parallel conductance after haemodynamic measurements. The blood was collected from the LV into a heparinized syringe and transferred into cuvettes to convert the conductance signal to volume using the catheter. The absolute volume of the rat was defined by calibrating the parallel conductance and the cuvette conductance.

### 2.9 Masson trichrome staining

Four weeks after treatment, the rats were euthanized, and the hearts were harvested. The hearts were fixed in 4% paraformaldehyde overnight, embedded in paraffin, and sectioned into 4 μm sections starting at the top of the apex using a microtome (Leica, RM2255, Germany). These sections were deparaffinized with xylene and fixed in Bouin’s solution at 56°C for 90 min. Next, the sections were stained with Weigert’s iron haematoxylin solution for 15 min at room temperature and washed with tap water for 15 min. Biebrich scarlet–acid fuchsin solution was then applied to the sections for 15 min at room temperature. Phosphomolybdic acid staining was performed for 15 min, followed by aniline blue staining for an additional 15 min. Between each step, the sections were washed. Finally, after the sections were mounted and imaged using Pannoramic MIDI, the percentage of fibrotic area relative to the entire LV wall area was quantified using ImageJ software with basic add-ons.

### 2.10 NRCM isolation

Neonatal rat hearts were collected at post-natal Day 2 (P2) and cut into small pieces. The minced tissue was transferred to 0.1% trypsin solution and incubated at 4°C overnight. After that, the trypsin was inhibited using media containing 10% FBS. The tissue was further digested with 1 mg/mL collagenase type 2 for 1 h and filtered using a cell strainer. The cells were centrifuged, and Percol was used to separate cardiomyocytes from cardiac fibroblasts. Neonatal rat cardiomyocytes (NRCMs) finally were washed and seeded on gelatin-coated dishes.

### 2.11 Cell survival assay

To measure cell survival after inducing inflammatory injury, the Cell Counting Kit 8 (CCK-8) was used. Cardiomyocytes or endothelial cells were seeded in 96-well plates at the density 10^4^ cells/well, with the medium containing lipopolysaccharide (LPS; Sigma, #L3129) or tumour necrosis factor alpha (TNF-α) (Sino Biological Inc., China, #10602-H01H), SCFAs at designed concentrations, and/or IKK2 inhibitor IV (Calbiochem, Sigma-Aldrich, #401481) at 18, 180, and 9 µM. Subsequently, CCK-8 was added to the wells and incubated for 2 h. The absorbance was measured using a microplate reader, and the optical density (OD) was converted to percentages based on untreated cells.

### 2.12 Western blot in NRCMs

NF-κB p65, IkBα, and their phosphorylated forms were detected in cell lysates through western blotting. Cells were lysed in RIPA buffer supplied with Roche cOmplete™ Protease Inhibitor Cocktail. Proteins were separated by 10% sodium dodecyl sulphate-polyacrylamide gel electrophoresis and transferred onto nitrocellulose membranes (Bio-Rad). The membranes were then blocked in TBS-T buffer (20 mM Tris-HCl, 150 mM NaCl, 0.1% Tween, pH = 7.6) containing 5% bovine serum albumin (BSA). Antibodies including anti-NF-κB p65 (Thermo Fisher Scientific, #436700), anti-NF-κB p65 phospho S536 (Abcam, ab76302), anti-IkBα (Abcam, ab32518), anti-IκBα phospho S36 (Abcam, ab133462), anti-phospho-IKKalpha/beta (16A6; Cell Signaling Technology, CST-2697S), anti-IKKalpha (Abcam, ab169743), and anti-GAPDH Loading Control (Invitrogen, # MA5-15738-HRP) were used to probe the protein on the membranes at the recommended concentration by the manufacturer. Immunoreactive bands were visualized using ECL Western Blotting Substrate (Bio-Rad). Coomassie blue G-250 staining was performed to visualize total protein as the loading control.

### 2.13 LDH assay

To determine the protective effects of SCFAs on endothelial cells against inflammatory injury, an lactate dehydrogenase (LDH) assay was employed. Briefly, cells were seeded in 96-well plates at the density 10^4^ cells/well with media containing LPS 20 μg/mL and SCFAs overnight. Then, the conditioned medium was transferred from each well to a new plate, and an LDH kit was added to the well. The absorbance was read using the microplate reader, and the OD values were converted to percentages representing dead cell percentage.

### 2.14 Immunocytochemistry

Immunofluorescence was performed as described in our published article. Briefly, cells were fixed with 4% PFA in PBS and blocked with 1% BSA in PBS for 60 min at room temperature. Cells were then incubated with primary antibodies diluted in PBS containing 1% BSA and 1% Tween-20 at 4°C overnight. The primary antibodies used in this study were carboxylate transporter [monocarboxylate transporter 1 (MCT1); Invitrogen #PA5-72957] and GPR41 (Invitrogen #PA5-75521), both diluted at a 1:50 ratio. After washing three times with 1% Tween-20 in PBS, the samples were incubated with a secondary antibody for 60 min at room temperature in the dark. Secondary antibodies used in this study include 1:500 anti-rabbit IgG Alexa Fluor 568 (Invitrogen #A11011). After washing again with 1% Tween-20 in PBS, the cells were stained with DAPI solution (VECTASHIELD) for nuclear staining and then mounted on slides. Imaging of the cells was performed using a Nikon A1HD25 high-speed and large field of view confocal microscope.

### 2.15 Neutrophil isolation

The collected blood was mixed with RPMI 1640 Medium at a 1:1 ratio. The mixture was layered onto Ficoll® Paque PLUS (GE17-1440-02) and centrifuged at 700 × *g* for 30 min at 20°C (0 acceleration, 0 deceleration). The upper layers of the density gradients were removed, leaving the bottom layer containing granulocytes and erythrocytes intact. Cells were washed with DPBS (Gibco™, 14190144), and then, 1× RBC lysis buffer (BioLegend, 420302) was added to lyse the red blood cells. Purified neutrophils were resuspended in cell-staining buffer (BioLegend, 420201), and FACS analysis was performed using anti-human CD11b BV605 (BioLegend, 301331), and anti-human CD66b-FITC (BioLegend, 984102) to validate the purity of neutrophils.

### 2.16 Neutrophil chemotaxis assay

Peripheral blood was obtained from healthy donors with full consent. The collection and processing were performed with the approval of the Human Subjects Ethics Sub-Committee (reference no.: 1-2021-29-F). Neutrophil isolation was described in detail in the Supplementary material. Neutrophil migration assays were conducted using QCM Chemotaxis Cell Migration Assay, 24-well (3 µm; Sigma-Aldrich, ECM505). Briefly, purified neutrophils were seeded into the migration chamber, and control medium or medium with SCFAs was added to the bottom wells as a chemoattractant for 1 h. Interleukin (IL)-8 was used as the positive control to induce neutrophil chemotaxis. Subsequently, migrated cells were post-stained with CyQUANT GR dye in cell lysis buffer for 15 min at room temperature. Fluorescence of migrated and invaded cells was read using BioTek Synergy™ H1 Microplate Reader at excitation/emission wavelength filter of 480/520 nm.

### 2.17 Macrophage culture and polarization

THP-1 cells (human monocytic cell line from acute monocytic leukaemia) were maintained in RPMI medium supplemented with 10% FBS and 1% Pen-Strep. The cells were maintained in an incubator at 37°C with 5% CO_2_. THP-1 cells were differentiated into M0 macrophages with 100 nM PMA for 72 h, followed by 24 h rest in PMA-free medium. M0 macrophages were polarized using 10 ng/mL LPS and 25 ng/mL for 72 h to obtain M1 macrophages. To obtain M2 macrophages, M0 macrophages were treated with 20 ng/mL IL-4 and 20 ng/mL IL-13 for 72 h. M0 macrophages were treated using SCFAs under M1/M2 conditions simultaneously for 24, 48, and 72 h accordingly.

### 2.18 Reverse transcriptase–qPCR

M0 macrophages were incubated with SCFAs under M1/M2 conditions for 24, 48, and 72 h accordingly. M1/M2 macrophages without SCFAs were used as positive controls, and untreated M0 macrophages were used as baseline control. RNA was isolated in TRIzol based on the manufacturer’s instruction (Invitrogen, 15596026). Collected RNA was transcribed into cDNA using the PrimeScript RT Master Mix Kit (Takara Bio, Inc.). Quantitative PCR (qPCR) was performed using PowerUp™ SYBR™ Green Mix (Applied Biosystems™, A25742). GAPDH was used as the reference gene for normalization, and relative changes in gene expression were determined using 2^−ΔΔCt^ method. Primer sequences used for qPCR are shown in Supplementary material online, *Table S1*.

### 2.19 Cytokine array

Cell-free supernatants from macrophages were collected after 72 h of incubation and assessed for cytokine expression using a semi-quantitative immunosorbent approach with Proteome Profiler Human Cytokine Array Kit/ARY005 (R&D Systems). Proteome profiling was performed in accordance with the manufacturer’s guidelines.

### 2.20 Statistical analysis

All quantitative data are shown as means ± SEM unless otherwise indicated. Statistical differences between the two groups were analysed using a two-tailed Student’s *t*-test. Significant differences between three or more groups were also analysed by analysis of variance (ANOVA) with Bonferroni’s *post hoc* analysis. Results were considered significant when the *P*-value was <0.05.

## 3. Results

### 3.1 Genetically modified probiotics efficiently produce SCFAs

**Figure 1:**
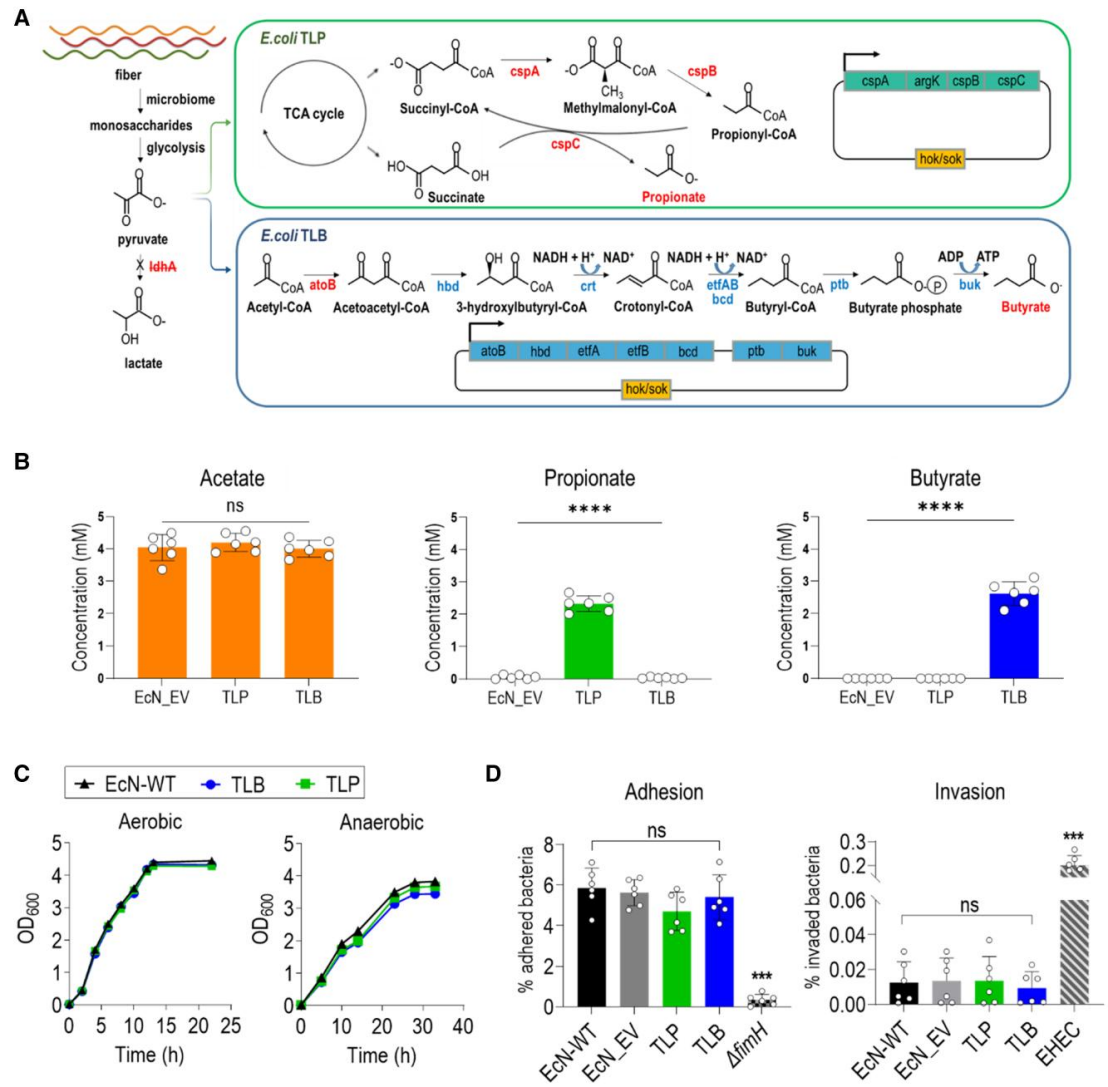
Generation of engineered EcN producing propionate and butyrate. (*A*) The major metabolic pathways and metabolic engineering strategies employed to develop two engineered EcN TLP and TLB strains. In the diagrams, *ldhA*, *cspA*, *cspB*, *cspC*, and *atoB* are endogenous genes. *Hbd*, *crt*, *etfAB*, *bcd*, *ptb*, and *buk* are exogenous genes. Black crosses indicate deleted genes. The genes were arranged as operons on plasmids carrying the stability system hok/sok. (*B*) Measurement of SCFAs in the culture medium of EcN-WT, TLP, and TLB was performed by using GC-MS analysis. Six single, fresh colonies on agar plates were inoculated into LB medium and cultured overnight at 37°C. The starter was then inoculated at a ratio of 1:100 into fresh BHI medium and cultured anaerobically. The medium was collected after 28 h for GC-MS analysis. The data are represented as mean ± SEM. *n* = 6 biologically independent samples per group. The data are represented as mean ± SEM. ****P < 0.0001 compared with EcN-WT. One-way ANOVA was used for statistical analyses. (*C*) Growth of EcN-WT, TLP, and TLB in BHI medium in aerobic conditions (37°C, shaking) and anaerobic conditions (37°C, anaerobic jar). Bacteria from a single colony of each strain were cultured under the indicated conditions, and growth was measured by OD at 600 nm. No statistically significant differences were detected. (*D*) Virulence assessment of the engineered *E. coli* in adhesion and invasion into intestinal Caco-2 cells. An invasive enterohaemorrhagic *E. coli* served as a positive control. The data are represented as mean ± SEM. ***P < 0.001 compared with EcN-WT. *n* = 6 biologically independent samples per group. One-way ANOVA was used for statistical analyses. ldhA, lactate dehydrogenase A; cspA, methylmalonyl-CoA mutase; cspB, methylmalonyl-CoA decarboxylase; cspC, propionyl-CoA::succinate transferase; argK, arginine kinase; atoB, acetyl-CoA acetyltransferase; BHBD, β-hydroxybutyryl-CoA dehydrogenase; crt, crotonase; etfAB, quinone oxidoreductases; bcd, butyryl-CoA dehydrogenase; ptb, phosphotransbutyrylase; buk, butyrate kinase; ns, not statistically significant.

Wild-type EcN does not efficiently produce propionate and butyrate. We, therefore, introduced separate biosynthesis pathways for propionate and butyrate into the bacteria. The genetic cassette of the *sbm*, which is responsible for propionate biosynthesis and carries the *cspA*, *cspB*, *cspC*, and *argK* genes from commensal *E. coli* K-12,^27^ was cloned into the pTLP plasmid. This plasmid was then transformed into EcN to enhance propionate production. For butyrate production, a heterologous pathway was introduced into the bacteria using the pTLB plasmid. This plasmid contained *atoB* from *E. coli* K-12, *BHBD*, *crt*, *etfAB*, and *bcd* from *C. butyricum* and *ptb* and *buk* from *C. acetobutyricum* (*Figure 1A*). The genes cloned from *C. butyricum* and *C. acetobutyricum* have previously been demonstrated to efficiently produce butyrate in *E. coli*.^28,29^ Since pyruvate is the precursor of both pathways, the pTLP and pTLB plasmids were introduced separately into EcN to prevent competition for pyruvate within a single strain to guarantee the efficient production of propionate and butyrate. Additionally, the *hok/sok* system was included to maintain the plasmids in the bacteria (see Supplementary material online, *Figure S1*).

To optimize SCFA production by channelling carbon flux into the designated pathways, various competing genes at the pyruvate and acetyl-CoA nodes were knocked out from the EcN genome (see Supplementary material online, *Figure S2A*). As shown in *Figure 1B*, the null mutant of *ldhA*, in the presence of either TLP or TLB plasmids, produced a significant amount of propionate (EcN:Δ*ldhA/*pTLP: 2.5 ± 0.17 mM) and butyrate (EcN:Δ*ldhA/*pTLB: 2.87 ± 0.24 mM) without affecting growth under both aerobic and anaerobic conditions (*Figure 1C*). In contrast, other null mutants such as Δ*adhE*, Δ*frd*, Δ*ackA*, and Δ*pta* showed reduced anaerobic growth (see Supplementary material online, *Figure S2B* and *C*), rendering them unsuitable for *in vivo* studies. To ensure the safety of these genetically modified bacteria, their virulence was compared with that of wild-type EcN using an *in vitro* Caco-2 cell model (intestine epithelial cells). As shown in *Figure 1D*, no significant difference in adhesion and invasion was observed between the wild-type and the engineered probiotics. To simplify the nomenclature of the different strains, EcN:Δ*ldhA/*pTLP and EcN:Δ*ldhA/*pTLB were designated as TLP and TLB, respectively. Moreover, a 1:1 mass ratio mixture of TLP and TLB, named EcN_TL, was used for studying the effect of SCFAs in the animal models. EcN transduced with empty vector (EcN_EV) was used as a control.

### 3.2 EcN_TL administration enhances plasma SCFA level

**Figure 2:**
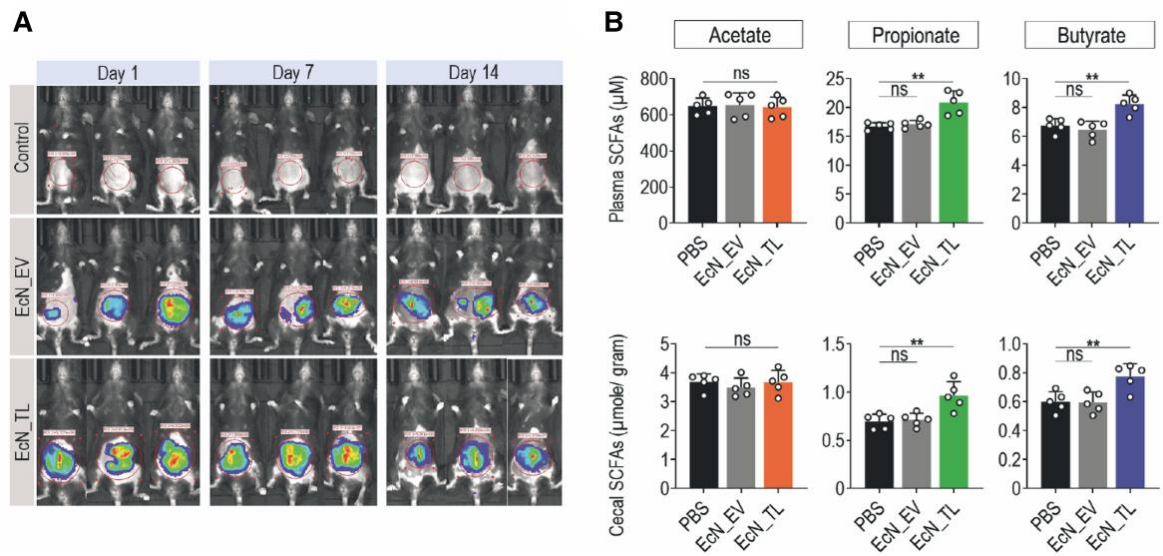
EcN_TL formula improved SCFA level *in vivo*. (*A*) Residence of bacteria in mouse gut visualized by luminescent intensity from the whole animal after antibiotic pre-treatment followed by daily administration of EcN_EV (EcN:ΔldhA is carrying empty vector) and EcN_TL (mixture of TLP and TLB in ratio 1:1). The mice were pre-treated with an antibiotic cocktail (vancomycin 0.125 g/L, neomycin 0.25 g/L, ampicillin 0.25 g/L, and metronidazole 0.25 g/L) in drinking water for 1 week (ABX). After 14 daily doses of probiotics (10^9^ cfu/dose), the mice were sacrificed. The plasma and cecum were collected for SCFA measurements by GC-MS. (*B*) SCFA concentrations in plasma and cecum. Graphs represent changes in individual SCFAs (acetate, propionate, and butyrate). The data are represented as mean ± SEM. **P < 0.01 compared with PBS control group; *n* = 5 biologically independent samples per group. One-way ANOVA was used for statistical analyses.

We next assessed the colonization of EcN_TL and subsequent secretion of SCFA *in vivo*. We introduced the luminescence cassette *LuxCDABE* from *P. luminescens* to both EcN_TL and EcN_EV to allow *in vivo* monitoring of bacterial colonization in the gut using the IVIS. To overcome the resistance of human-origin probiotics in the mouse gut (see Supplementary material online, *Figure S3*), mice were pre-treated by an antibiotic cocktail (ABX).^12,30^ Daily feeding of EcN_TL and EcN_EV for 14 days showed stable colonization of bacteria in the gut (*Figure 2A*). We then measured the concentration of in the plasma. The plasma propionate concentration in the EcN_TL-fed group was 24.66% higher than that in the PBS-fed and EcN_EV-treated groups (*P* < 0.01). Similarly, EcN_TL improved butyrate concentration in the plasma by 22% compared with other treatment groups (*P* < 0.01; *Figure 2B*). This enhancement was maintained after cessation of treatment, as the measurement was taken 24 h after the last dose of probiotics. It is worth noting that 7 days of daily feeding with EcN_TL only increased the propionate plasma concentration but not butyrate (see Supplementary material online, *Figure S4*).

**Figure 3:**
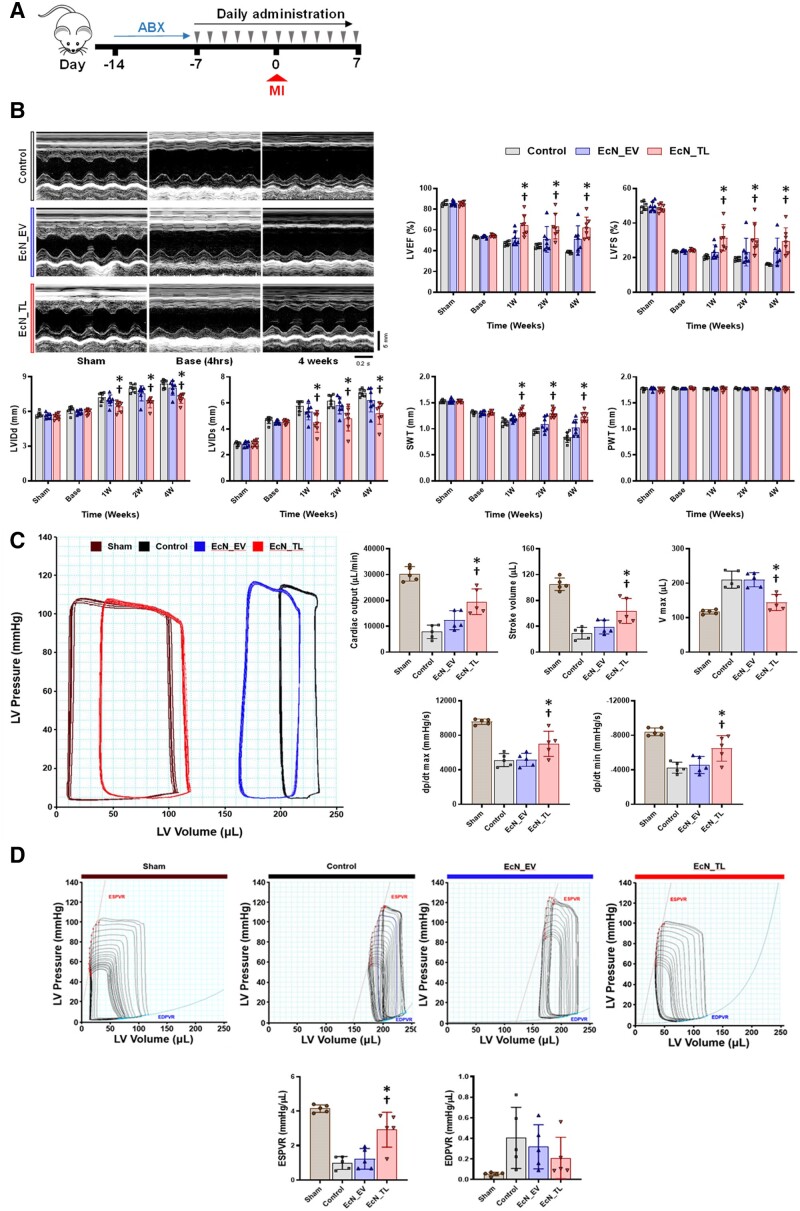
Effect of EcN_TL on improving cardiac function after MI. (*A*) Schematic of EcN_TL administration in animals. (*B*) Representative images M-mode of three groups at 1 and 4 weeks post-I/R and the measurement of LV EF, left FS, LV internal diameter at end-diastole (LVIDd), LV internal diameter at end-systole (LVIDs), septal wall thickness (SWT), and posterior wall thickness (PWT). The data are represented as mean ± SEM. *P < 0.05 vs. control. ^†^*P* < 0.05 vs. EcN_EV. *n* = 7. The experiment was performed with seven animals for each group. Statistical differences between three groups were examined by two-way ANOVA followed by Bonferroni’s *post hoc* analysis (*P < 0.05 vs. control. ^†^*P* < 0.05 vs. EcN_EV). (*C*) Representative images of the haemodynamic PV curve on steady state at 4 weeks post-I/R injury and the measurement of cardiac output, stroke volume, volume max (*V*_max_) at end-diastole, maximal rate of pressure changes during systole (dP/dt_max_), and minimal rate of pressure changes during diastole (dP/dt_min_). The data are represented as mean ± SEM. *P < 0.05 vs. control. ^†^*P* < 0.05 vs. EcN_EV. *n* = 5 biologically independent animals per group. One-way ANOVA was used for statistical analyses. (*D*) The slope of ESPVR indicating the intrinsic cardiac contractibility as measured by transient inferior vena cava (IVC) occlusion. Slope of EDPVR. In *C* and *D*, the experiment was performed with five animals for each group. Data are shown as the mean ± SEM. Statistical differences between three groups were examined by one-way ANOVA followed by Bonferroni’s *post hoc* analysis (*P < 0.05 vs. control. ^†^<0.05 vs. EcN_EV).

We optimized the probiotic regimen in rats in order to investigate the preventive effects of EcN_TL against myocardial I/R injury in the well-established rat model. Daily oral administration of PBS, EcN_EV, or EcN_TL for 14 days resulted in a similar enhancement of SCFA concentration in the blood of the rats (see Supplementary material online, *Figure S5*). After the initial 7 days of daily oral administration, on Day 7, I/R injury was induced by ligating the LAD coronary artery. Subsequently, the animals received an additional 7 daily doses of the treatment (*Figure 3A*). Serial echocardiography was performed at baseline, 1 week, 2 weeks, and 4 weeks after induction of I/R injury to evaluate the protective effect of EcN_TL. M-mode images showed that EcN_TL remarkably enhanced contractile activity and thickness in the left anterior wall after I/R, as compared with both the PBS-fed groups (control) and EcN_EV groups (see Supplementary material online, *Figure S3B* and *S6*). EcN_TL promoted EF and FS by 64.6% and 31.5%, respectively, at 1-week post-I/R (*P* < 0.05). Additionally, EcN_TL groups exhibited lower LV internal diastolic dimension and LV internal systolic dimension (*P* < 0.05), as well as higher septal wall thickness compared with the other experimental groups (*P* < 0.05; *Figure 3B*). Furthermore, we conducted PV loop analysis to directly assess cardiac function. Haemodynamic criteria such as cardiac output, cardiac stroke, *V*_max_, and dP/dt max/min demonstrated significant enhancement of LV function in the EcN_TL groups (*P* < 0.05; *Figure 3C*). The EcN_TL-fed rats also exhibited a 1.9-fold higher ESPVR and a trend of reduction in the slope of the EDPVR compared with both control and EcN_EV-fed groups (*P* < 0.05), indicating improved intrinsic cardiac contractibility as it represents the maximal pressure generated by the LV at any given volume (*Figure 3D*). Collectively, these results clearly indicated that pre-treatment with EcN_TL protected the heart from myocardial I/R injury, significantly improved heart function, and attenuated adverse LV remodelling following I/R injury.

**Figure 4:**
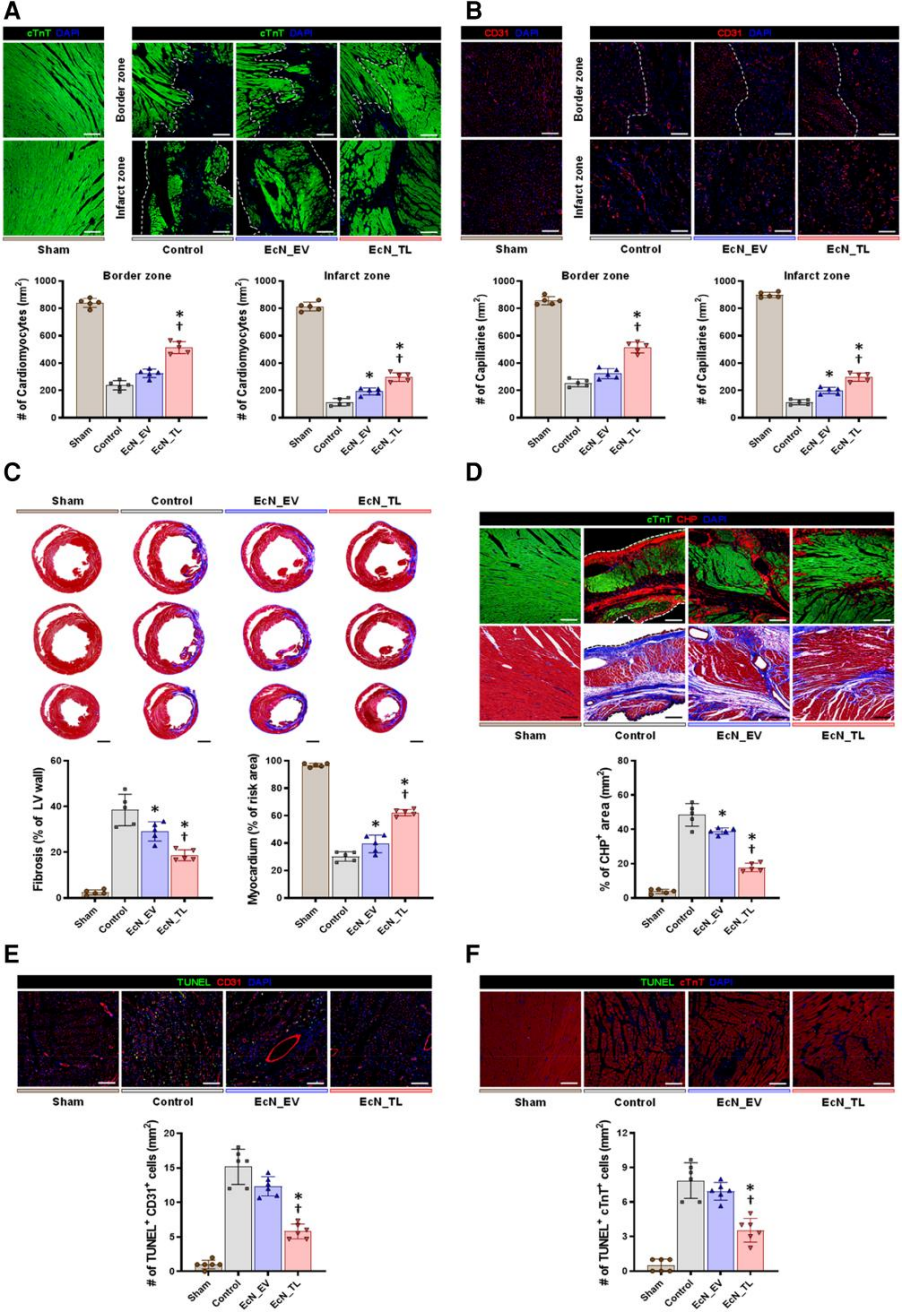
The effect of EcN_TL on improving the microenvironment of the damaged heart. (*A*) Representative images of cardiomyocytes stained with cTnT (green) on the infarct zone and border zone at 4 weeks and the quantification summary. *n* = 5. Scale bars: 100 µm. (*B*) Representative images of capillaries stained with CD31 (red) on the infarct zone and border zone at 4 weeks and the quantification summary. *n* = 5. Scale bars: 100 µm. (*C*) Representative images of Masson’s trichrome staining at 4 weeks and quantification summary of a percentage of fibrosis and viable myocardium. *n* = 5. Scale bars: 2000 µm. (*D*) Representative images of denatured collagen stained with CHP (red) and cardiomyocytes (green) on the infarct zone at 4 weeks and the quantification summary. *n* = 5. Scale bars: 100 µm. In *A*–*D*, the experiment was performed with five heart tissue for each group. Data are shown as the mean ± SEM. Statistical differences between three groups were examined by one-way ANOVA followed by Bonferroni’s *post hoc* analysis (*P < 0.05 vs. control. ^†^*P* < 0.05 vs. EcN_EV). (*E*) Representative images of apoptotic CMs in infarct zone 3 days after MI induction and quantification summary. TUNEL (green), cTnT (red), and DAPI (blue). *n* = 6. Scale bars: 100 µm. (*F*) Representative images of apoptotic ECs in infarct zone 3 days after MI induction and quantification summary. TUNEL (green), CD31 (red), and DAPI (blue). *n* = 6. Scale bars: 100 µm. In *E* and *F*, the experiment was performed with six heart tissues for each group. Data are shown as the mean ± SEM. Statistical differences between three groups were examined by one-way ANOVA followed by Bonferroni’s *post hoc* analysis (*P < 0.05 vs. control. ^†^*P* < 0.05 vs. EcN_EV).

Next, we examined the morphological and pathological changes in the hearts of the control, EcN_EV, and EcN_TL groups. We first assessed the number of viable cardiomyocytes as determined by troponin T (TnT, a cardiomyocyte-specific marker) staining. In both the infarct and border zones of the hearts in the EcN_TL-fed rats, there were significantly more viable cardiomyocytes than in other groups (*P* < 0.05; *Figure 4A*). Conversely, the number of TUNEL-positive cells, indicating apoptotic cells in the infarct area, was significantly lower in the EcN_TL group (*Figure 4E* and *F*). Furthermore, the number of small blood vessels, primarily capillaries positive for the endothelial cell marker CD31, in both the border and infarct zones, was greater in the EcN_TL-fed group compared with the other two groups (*Figure 4B*). Additionally, Masson’s trichrome staining used to quantify cardiac fibrosis showed remarkably lower collagen (blue) and wider viable myocardium (red) in EcN_TL groups compared with both the control and EcN_EV groups (*Figure 4C*). Lastly, hybridizing peptide (CHP) staining, which detects degraded collagen, revealed that EcN_TL effectively reduced collagen degradation in the infarct zone compared with the control and EcN_EV groups (*P* < 0.05; *Figure 4D*). These findings were particularly significant because during the acute inflammatory phase post-MI, infiltrating leukocytes phagocytose cell debris and exhibit proteolytic activity. Collagen degradation by these proteolytic enzymes from leukocytes contributes to tissue damage during inflammation. In this regard, our results suggested that EcN_TL may limit the recruitment of inflammatory cells and inflammation-induced myocardial tissue damage.

**Figure 5:**
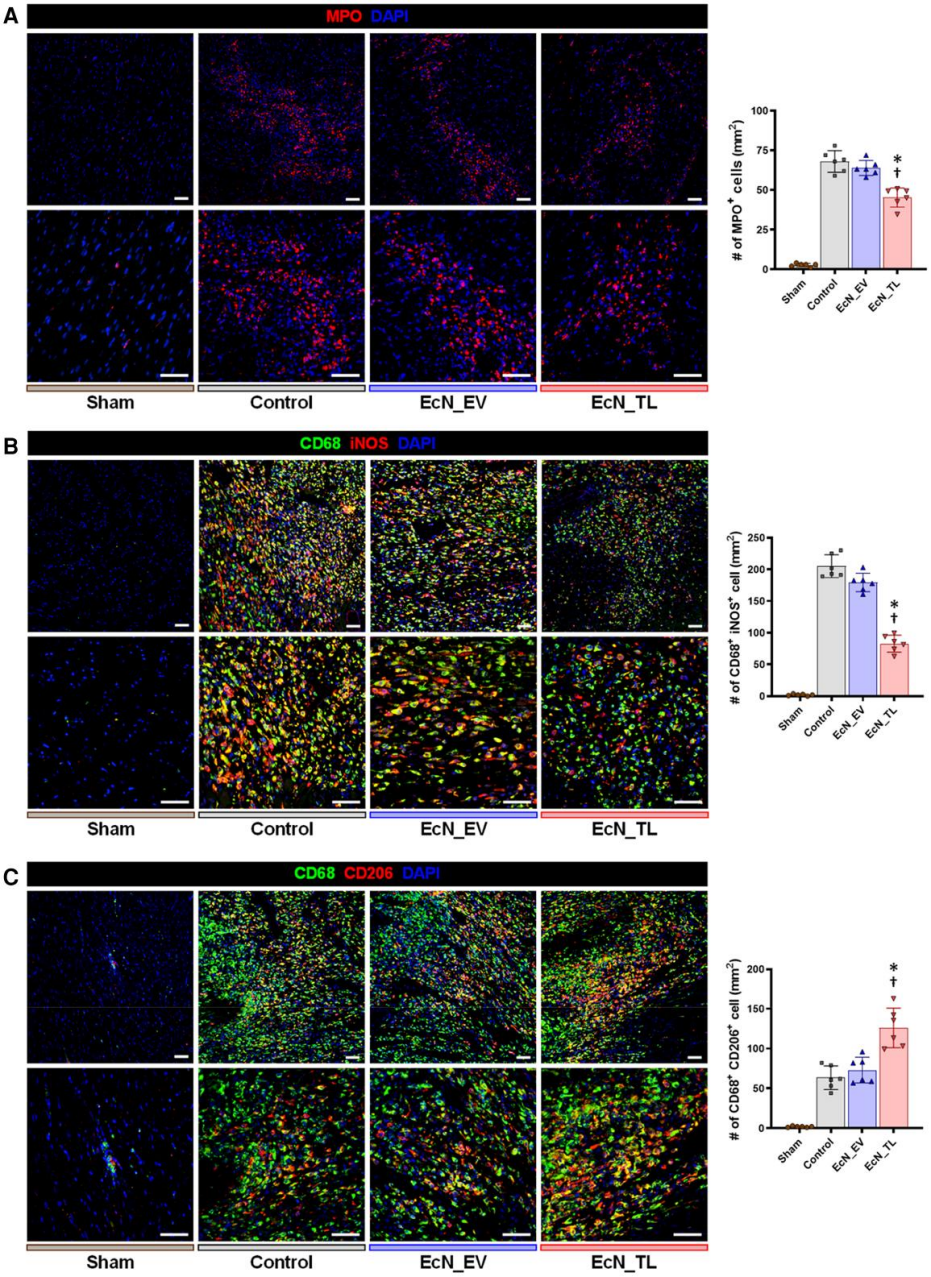
Inhibition of acute inflammatory reactions of EcN_TL. (*A*) Representative images of neutrophils stained with MPO in the infarct zone 3 days after MI induction and the quantification summary. MPO (red) and DAPI (blue). *n* = 6. Scale bars: 50 µm. (*B*) Representative images of M1 macrophages in the infarct zone 3 days after MI induction and the quantification summary. CD68 (green), iNOS (red), and DAPI (blue). *n* = 6. Scale bars: 50 µm. (*C*) Representative images of M2 macrophages in infarct zone 3 days after MI induction and quantification summary. CD68 (green), CD206 (red), and DAPI (blue). *n* = 6. Scale bars: 50 µm. In *A*–*C*, the experiment was performed with six heart tissues for each group. Data are shown as the mean ± SEM. Statistical differences between three groups were examined by one-way ANOVA followed by Bonferroni’s *post hoc* analysis. *P < 0.05 vs. control. ^†^*P* < 0.05 vs. EcN_EV.

Considering that SCFAs have demonstrated significant anti-inflammatory effects in other studies, together with our results suggesting reduced inflammation-induced myocardial damage, we hypothesized that EcN_TL protects the heart from I/R injury through its anti-inflammatory effects. To assess systemic inflammation, we analysed blood collected from the rats post-I/R injury on Days 3 and 7. We found that the percentage of neutrophils, an indicator of systemic inflammation and a potential marker of cardiac injury,^31^ was reduced in the EcN_TL-fed group compared with the other groups (see Supplementary material online, *Figure S7*). Furthermore, immune staining in heart tissues harvested 4 weeks after I/R injury revealed that the number of neutrophils, detected by MPO staining, was significantly lower in the EcN_TL-fed group compared with the other groups (*Figure 5A*). Interestingly, a lower number of iNOS^+^ M1 macrophages and a higher number of CD206^+^ M2 macrophages were detected in EcN_TL-treated hearts compared with the other groups (*Figure 5B* and *C*). The recruitment of different phenotypes of macrophages and their subsequent actions plays a crucial role in the innate immune response following myocardial injury. During the acute inflammatory phase of MI, the initial wave of infiltrated macrophages promotes clearance of cellular debris and releases pro-inflammatory cytokines, further amplifying inflammation. In the later stages, macrophages transition an anti-inflammatory ‘M2’ phenotype and participate in the inflammation resolution and healing.^32^ Taken together, these results evidently indicated that EcN_TL feeding provided extensive benefits, particularly by protecting the heart from I/R injury through its anti-inflammatory effects.

### 3.3 SCFAs protect cardiomyocytes against inflammation-induced injury via NF-κB pathway

**Figure 6:**
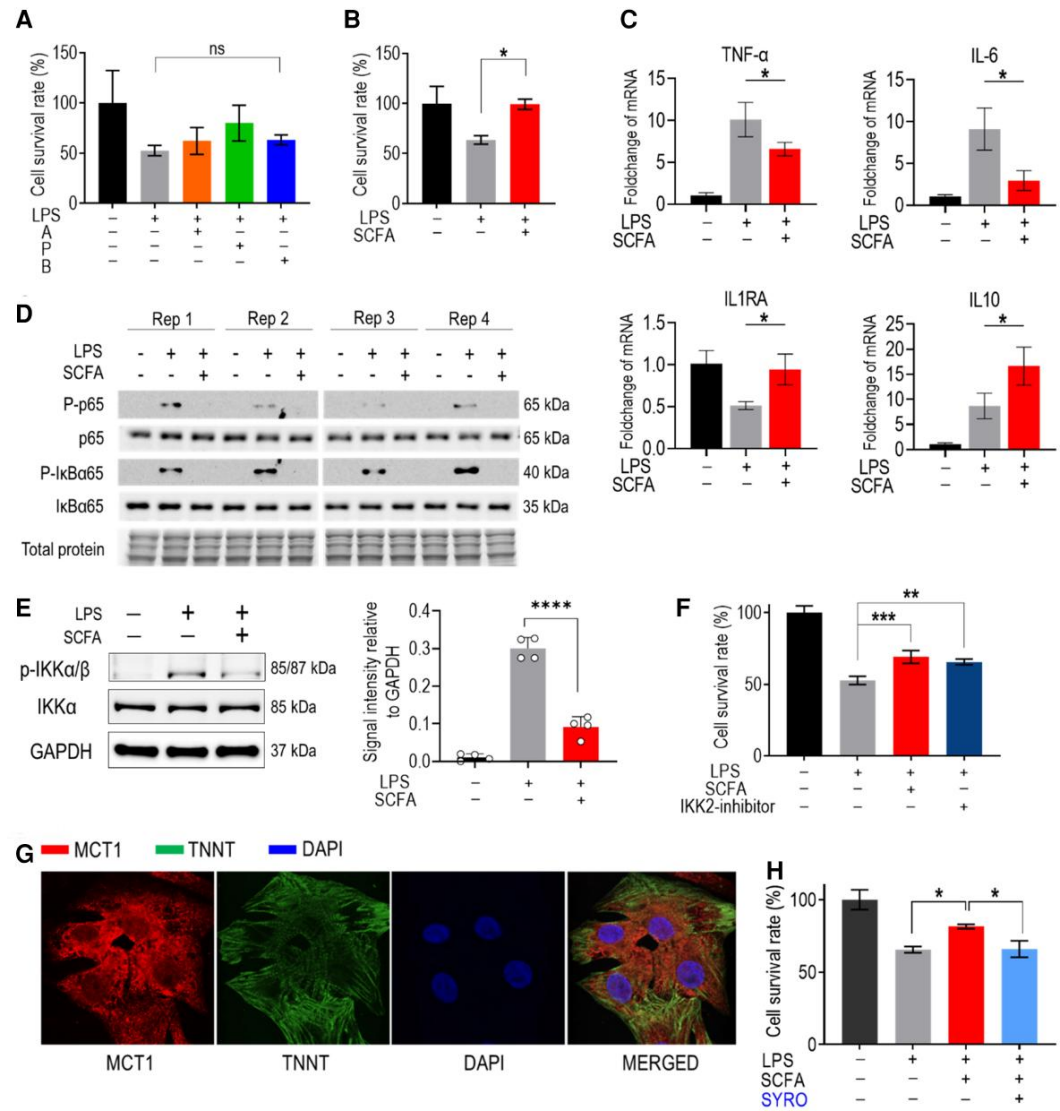
SCFAs protect cardiomyocytes during inflammation. (*A* and *B*) Combination of SCFAs improves cardiomyocyte survival under inflammatory injury. Cardiomyocytes were incubated with individual SCFAs in which acetate, propionate, and butyrate are indicated as A, P, and B in the graph, respectively (*A*) and mixture on SCFAs (500 μM acetate, 15 μM propionate, and 5 μM butyrate), which is indicated as SCFAs in the graph (*B*) for 18 h followed with a treatment of 10μg/mL LPS for 16 h. Cell survival was determined by the Cell Counting Kit-8 assay. Data are shown as the mean ± SEM. *P < 0.05. *n* = 3 biologically independent samples per group. One-way ANOVA was used for statistical analyses. (*C*) qPCR of pro-inflammatory cytokine and anti-inflammatory cytokine gene expression in cardiomyocytes during inflammation triggered by LPS. Cardiomyocytes were incubated with the mixture of SCFAs (500 µM acetate, 15 µM propionate, and 5 µM butyrate) for 18 h followed by a treatment of 10 µg/mL LPS for 15 min. Data are shown as the mean ± SEM. *P < 0.05. *n* = 3 biologically independent samples per group. One-way ANOVA was used for statistical analyses. (*D*) SCFA treatment inhibits phosphorylation of NF-κB and IκBα in cardiomyocytes. The level of total NF-κB P65, S536 phosphorylated P65, and IκBαS36 phosphorylated IκBα was detected by western blot. The total protein served as a loading control. (*E*) SCFA treatment inhibits phosphorylation of IKKα/β in cardiomyocytes. The level of S176/180 phosphorylated IKKα/β and total IKKα were detected by western blot. The GAPDH protein served as a loading control. The western blot was performed with four replications and a representative image was shown. The abundance of protein was determined from the band intensity using ImageJ software, normalized relative to the GAPDH protein control and plot to the bar graph. Data are shown as the mean ± SEM. *P < 0.05. *n* = 4. Statistical differences between the two groups were examined by unpaired Student’s *t*-test. (*F*) SCFA acted as NF-κB inhibitors. When IKK2 (an inhibitor of NF-κB) was added, SCFA lost the protective effects on cardiomyocytes under inflammation injury. Cell survival was determined by the Cell Counting Kit-8 assay. Statistical differences between the two groups were examined by unpaired Student’s *t*-test. *P < 0.05. *n* = 4. (*G* and *H*) SCFAs entered cardiomyocytes through MCT1. (*G*) Immunocytochemistry of cardiomyocytes stained with MCT1. MCT1 expression (red) can be detected on the cell membrane. Cytoplasm and nucleus were stained by troponin T TNNT (green) and DAPI (blue). (*H*) An MCT1 inhibitor, syrosingopine, abolishes SCFA protection effect. The SCFA and LPS treatment was similar to survival assay. Syrosingopine was added along with SCFAs. *P < 0.05. *n* = 4 for non-treated control and *n* = 3 for other groups. One-way ANOVA was used for statistical analyses.

We then investigated the molecular mechanism underlying SCFA-induced cardioprotection. While feeding EcN_TL prevented ischaemic injury during I/R, we discovered that SCFAs did not directly protect cardiomyocytes from I/R injury. *In vitro* experiments using H9C2 myoblasts and primary rat neonatal cardiomyocytes exposed to H_2_O_2_ to simulate I/R injury showed that neither individual SCFAs (acetate, propionate, and butyrate) nor their combination provided protection (see Supplementary material online, *Figure S8*). We next examined the effect of SCFAs on inflammation-induced injury in cardiomyocytes. Interestingly, only the combination of SCFAs, but not individually, significantly enhanced the survival of cardiomyocytes subjected to LPS-induced cell injury (*Figure 6A* and *B*). Moreover, SCFA treatment significantly reduced the mRNA levels of inflammatory cytokines, including TNF-α and IL-6, while promoting the expression of anti-inflammatory cytokines like IL1RA and IL-10 (*Figure 6C*).

To investigate the molecular mechanism of SCFAs in cardioprotection, we assessed the activation of NF-κB, a central regulator of inflammation that mediates numerous pro-inflammatory gene expressions, including TNF-α, IL-6, and IL-1β.^33^ By examining the phosphorylation of p65 and IκBα, we observed that LPS effectively activated NF-κB in cardiomyocytes (*Figure 6D* and *E*). However, consistent with a previous study showing that SCFAs inhibit NF-κB pathway in cells expressing SCFA-sensing receptors such as GPR41 and GPR43,^34^ co-treatment of LPS with SCFAs inhibited the phosphorylation of p65, IκBα, and IKKα/β without altering the total protein levels. Subsequently, the addition of an IKK2 inhibitor in the cultured cardiomyocytes significantly abolished the protective effects of SCFA (*Figure 6F* and Supplementary material online, *Figures S9–S11*).

We then sought to identify a cardiomyocyte-specific membrane protein that responds to SCFA effects. No known SCFA receptor was found to be expressed in cardiomyocyte.^35^ Since SCFAs affect the cells through both membrane receptors and intracellular mechanisms, we tested MCT1, a SCFA transporter, as the potential candidate.^36^ NRCMs were found to express MCT1 on the cell surface as shown by immunohistochemical staining (*Figure 6G*). Treatment with an MCT1 inhibitor, syrosingopine,^37^ abolished the aforementioned protection effect of SCFAs on cardiomyocytes (*Figure 6H*). These data strongly suggest that MCT1 is a potential SCFA receptor in cardiomyocytes that plays a critical role in SCFA-mediated cardiac protection.

### 3.4 SCFAs exhibited anti-inflammation in immune cells

**Figure 7:**
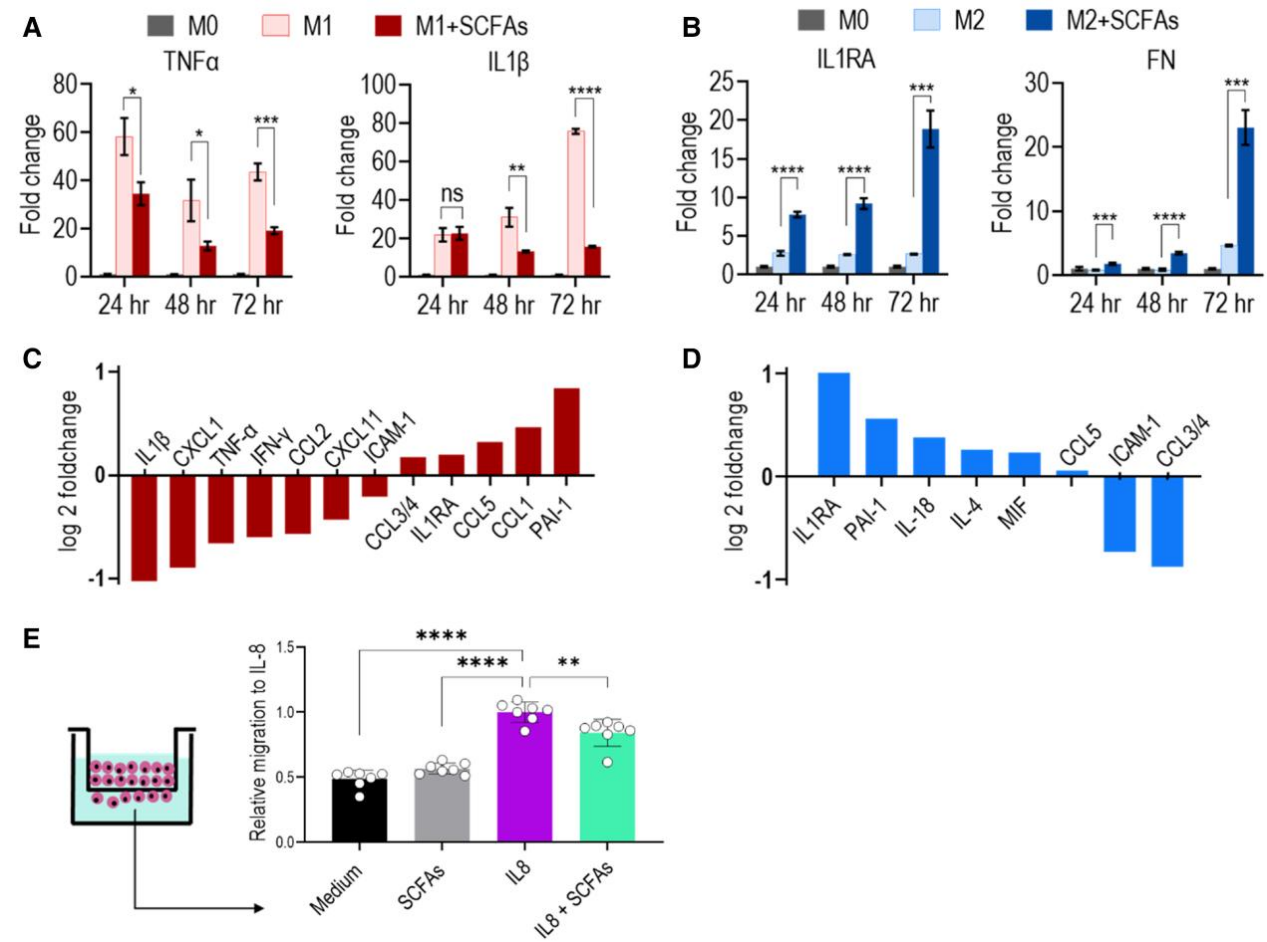
Effect of SCFAs on innate immune cells. (*A* and *B*) SCFAs drive anti-inflammatory properties in macrophages under both M1 and M2 polarizations. Measurement of cytokine production at mRNA level by qPCR for THP-1-derived M0 macrophages polarized into M1 or M2 macrophage phenotype in the presence and absence of SCFAs. Non-polarized M0 macrophages were used as the baseline control. (*A*) Pro-inflammatory cytokines TNFα and IL-1β were down-regulated in the presence of SCFAs under M1 condition. (*B*) Anti-inflammatory cytokine IL1RA and wound healing extracellular matrix FN were up-regulated by SCFAs under M2 condition. Data are shown as the mean ± SEM. *P < 0.05. *n* = 3 biologically independent samples per group. One-way ANOVA was used for statistical analyses. (*C* and *D*) Cytokine array for supernatant in macrophage culture showing log2 fold changes on secreted cytokines in M1 (*C*) and M2 (*D*) macrophages in the presence of SCFAs to that of M1 and M2 only controls without SCFAs. (*E*) SCFAs inhibit neutrophil migration. Migration assay for neutrophils in the presence of SCFAs and/or chemoattractant IL-8 in the transwell system. The freshly isolated neutrophils from blood were maintained in 1640 medium with 10% FBS and 5 × 10^5^ neutrophils were added to the upper compartment in a 24-well plate. IL-8 (10 ng/mL) was used as the chemoattractant (positive control). Mixed SCFAs (500 μM acetate, 15 μM propionate, and 5 μM butyrate) were used in this assay. Migrated neutrophils were quantified through cell lysis and detection using Cy-QUANT dye (DNA dye) by quantifying endpoint fluorescent intensities for all replicates. Data are shown as the mean ± SEM. *P < 0.05. *n* = 7 biologically independent samples per group. One-way ANOVA was used for statistical analyses.

Macrophages and neutrophils are both crucial components of the inflammatory response. In the MI experiments, we observed a decrease in the ratio of M1 to M2 macrophages at the infarct site in EcN_TL-fed rats, suggesting that SFCAs may influence macrophage polarization. Therefore, we investigated the impact of SCFAs in M1 vs. M2 macrophage polarization. The human THP-1–differentiated M0 macrophages were differentiated into M1 or M2 phenotype in the presence of a mixture of SCFAs, including acetate, propionate, and butyrate. During M1 differentiation, SCFA treatment suppressed the expression of inflammatory cytokines TNF-α and IL-1β (*Figure 7A*), indicating a dampening effect on the inflammatory phenotype of M1 macrophages. In contrast, SCFA treatment enhanced the expression of IL1Ra, an IL-1 antagonist known to reduce inflammation. The expression of fibronectin-1 (FN1), important for the wound healing function of M2 macrophages, was also elevated upon SCFA treatment (*P* < 0.001; *Figure 7B* and Supplementary material online, *Figure S12*). To further investigate the broader effect of SCFAs on cytokine production, we utilized a cytokine array to analyse the protein levels of a panel of cytokines in the supernatant of M1- or M2-differentiated macrophages. SCFAs down-regulated the expression of pro-inflammatory cytokines (IL-1β, CXCL-1, TNF-α, IFN-γ, and CCL-2) in M1 macrophages, while stimulating the production of anti-inflammatory molecules (IL1RA, PAI-1, and IL-4) in M2 macrophages (*Figure 7C* and *D* and Supplementary material online, *Figure S13*). These findings indicated that SCFAs modulate macrophage polarization and function towards an anti-inflammatory phenotype.

Next, we examined the effect of SCFAs on neutrophil migration induced by IL-8, a chemokine expressed by cardiomyocytes after MI.^38^ In a modified Boyden chamber transwell system, neutrophils in the upper compartment migrated towards the chemoattractant IL-8 in the lower compartment, while medium or SCFAs alone induced minimal neutrophil chemotaxis. The presence of SCFAs at serum concentration inhibited IL-8-induced neutrophil migration, suggesting a direct effect of SCFAs on neutrophil chemotaxis (*Figure 7E*).

During inflammation, neutrophils bind to the adherent molecules highly expressed in endothelial and roll along the vessel wall, following chemoattractants released from the injured myocardium. We found that SCFAs protected human endothelial cells from inflammatory injury, suggesting that SCFAs dampen the inflammatory response in endothelial cells expressing the receptor GRP41 (see Supplementary material online, *Figure S14*).

Overall, our results demonstrated that the combination of acetate, propionate, and butyrate at physiological plasma ratio suppresses the inflammatory response in endothelial cells, inhibits IL-8-dependent neutrophil chemotaxis, reduces the pro-inflammatory M1 phenotype, and promotes the anti-inflammatory wound healing M2 macrophages.

## 4. Discussion

Due to the extremely limited regenerative potential, heart disease–related injuries inevitably result in irreversible and fatal damage to the heart. Consequently, apart from making lifestyle changes, the most promising approach to safeguard the heart from injury is through effective prevention strategies. However, currently available preventative strategies are largely inadequate and mostly ineffective. In our study, we successfully developed a novel strategy to prevent ischaemic heart disease by orally administering an engineered probiotic called EcN_TL. This probiotic continuously and consistently secretes a physiological level of SCFAs into the bloodstream. EcN_TL was created by introducing two biosynthetic pathways, propionate and butyrate, into the EcN strain (see Supplementary material online, *Figures S15* and *S16*). EcN is a short-lived probiotic isolated from the human gut and has been widely used for over a century to treat inflammatory bowel disease and irritable bowel syndrome.^39,40,41^ Its long history of safety, tolerability in the human body, knowledge of its molecular background, and availability of genetic manipulation tools make EcN an ideal candidate for the development of engineered therapies.^42^ In fact, a synthetic live bacterial therapeutic for the human metabolic disease phenylketonuria is currently in Phase 2 clinical trials, highlighting the potential and feasibility of using EcN as a bio-therapeutic.^43^ When fed to rats for 1 week, EcN_TL effectively protected the animals from subsequent myocardial I/R injury, leading to a significant improvement in LV heart function and ameliorating adverse cardiac remodelling and fibrosis. Moreover, the SCFAs protected the cardiomyocytes from cell death induced by inflammation, although they did not have a significant effect on ischaemic stress. Additionally, SCFAs exhibited anti-inflammatory effects on immune cells.

Consistent with previous studies that demonstrated significant anti-inflammatory effects of SCFAs on macrophages,^10,44,45^ we showed that oral treatment of EcN_TL exerted cardioprotective effects through both immune cell-dependent and cell-independent mechanisms, primarily by reducing inflammation. SCFAs directly suppressed pro-inflammatory cytokine production in cardiomyocytes by inhibiting the activation of NF-κB, a key transcription factor for the production of various pro-inflammatory cytokines that exacerbate adverse cardiac remodelling and contribute to heart failure.^9^ Indeed, NF-κB inhibition has been proposed as a promising target for treating MI.^46^ In addition to reducing pro-inflammatory cytokines in MI hearts, EcN_TL also up-regulated anti-inflammatory cytokines such as IL1RA and IL-10. Cardiomyocytes do not express any known SCFA receptor. We discovered that the effect of SCFAs is dependent on MCT1 expression, suggesting a potential role of MCT1 as a novel SCFA receptor.

SCFAs have been found to have a direct effect on immune cells. Previous studies have demonstrated that propionate targets regulatory T cells to modulate inflammatory reactions, alleviates T helper cell homeostasis, and reduces immune cell infiltration, which leads to reduced hypertensive cardiac damage and atherosclerosis.^7^ Our study focused on the effect of SCFAs on innate immune cells, which play a major role in initiating and modulating the inflammatory response. SCFAs promoted the polarization of macrophage towards the M2 phenotype, in both *in vivo* MI hearts and *in vitro* models. We observed an increase in the number of M2 macrophages and a decrease in the number of M1 macrophages at the infarct zone in MI hearts when animals were fed EcN_TL. Consistently, SCFA administration increased the level of anti-inflammatory molecules and decreased the level of pro-inflammatory cytokines in macrophages. Furthermore, SCFA treatment resulted in less neutrophil infiltration into the MI heart *in vivo* and inhibited neutrophil migration *in vitro*. These results strongly support the role of SCFAs in regulating macrophage polarization towards a wound healing phenotype, reducing neutrophil-induced inflammatory injury, and orchestrating a microenvironment that promotes tissue repair in the MI heart. Previous studies have also shown that butyrate modulates the expression of genes related to inflammation and apoptosis to maintain a balance in the gut–heart axis.^47,48^ Our study further expands the repertoire of the immune effects of SCFAs and elucidates the immune cell-dependent and cell-independent mechanisms by which SCFAs exert cardioprotective effects.

While diets supplemented with SCFAs have been shown to provide benefits in heart diseases prevention and protection,^6,7,49^ maintaining effective SCFA levels in the bloodstream remains a critical challenge. The concept of using probiotics to release SCFAs was introduced a decade ago, but an effective probiotic treatment for MI has not yet been developed.^50,51^ A recent study on the relationship between the microbiome and cardiac repair highlighted the role of SCFAs in post-MI recovery.^12^ Our probiotic, EcN_TL, possesses the ability to constantly release SCFAs and maintain their concentration in the bloodstream during acute MI, thereby reducing of cardiomyocyte death and fibrosis, improving contractibility of LV, and enhancing cardiac performance.

To develop a heart-protecting probiotic for future human use, we engineered EcN_TL with a plasmid-based system that includes a *hok/sok* system to maintain the plasmids without the use of antibiotics. Following the FDA Guidance on Live Biotherapeutic Organisms, which emphasizes genetic stability and the avoidance of antibiotic resistance genes, we are currently developing new generations of EcN_TL by integrating SCFA biosynthetic genes into the chromosomes of EcN. Importantly, the effective delivery of SCFAs by EcN_TL is independent of fibre intake. In other words, the prevention of heart disease mediated by our probiotics is independent of any diet or lifestyle changes. This makes EcN_TL a safe, convenient, and appealing SCFA-specific supplement.^52^

In conclusion, our results provided a strong basis for utilizing synthetic live bacterial therapeutics to prevent heart diseases and laid the foundation of engineered probiotics as bio-therapeutic agents that target the gut–heart axis. Beyond protecting against CHDs, our developed probiotic EcN_TL, which provides constant delivery of SCFAs, can be applied to other health conditions. As a tool, it can be used to probe the role of SCFAs in the bidirectional communication of gut-related axes.

**Translational perspective**

We report a promising approach to effectively prevent heart injury caused by ischaemic heart disease, including myocardial infarction (MI), by using novel engineered probiotics, namely EcN_TL, that continuously secrete short-chain fatty acids (SCFAs). We found that oral administration of EcN_TL maintained the physiological concentration of the SCFAs in the plasma. Importantly, daily administration of EcN_TL for 14 days before MI significantly reduced the MI-induced cardiac injury, evidenced by improved cardiac function and reduced adverse cardiac remodelling. Based on these results, we suggest that the engineered probiotics could serve as a convenient and safe preventive solution for individuals at high risk of MI. This also opens up a new avenue for probiotics-based therapy.

## Supplementary Material

cvae128_Supplementary_Data

## Data Availability

Data are available upon request to the corresponding author.
