# Supplementary material for: Daily oral administration of probiotics engineered to constantly secrete short-chain fatty acids effectively prevents myocardial injury from subsequent ischaemic heart disease
Source: Cardiovasc Res. 2024 Jun 8;120(14):1737–51. doi: 10.1093/cvr/cvae128 (PMC11587561; doi:10.1093/cvr/cvae128)
Supplement: cvae128_Supplementary_Data [file cvae128_supplementary_data.docx]

**Figure S1**

**
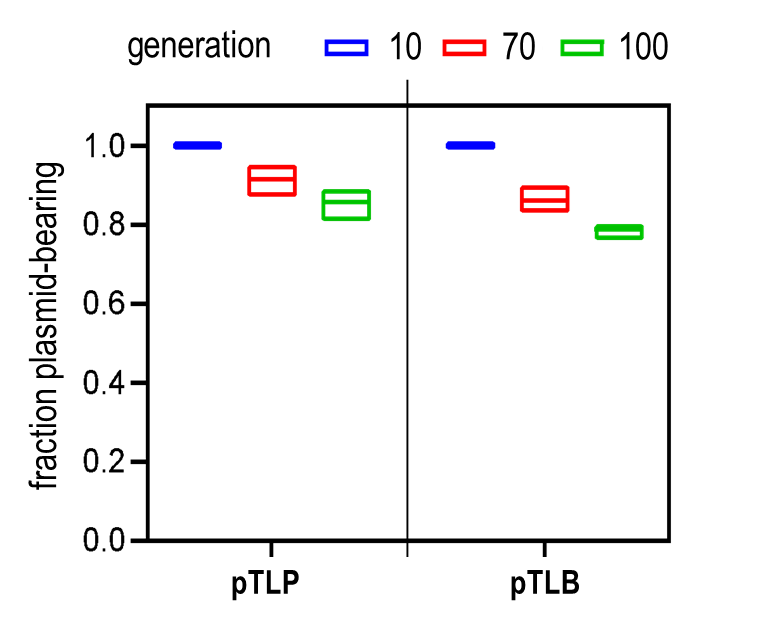
**

**Figure S1. Stability of pTLP and pTLB plasmids in EcN through 100 generations**. A plasmid stability assay was performed on EcN:Δ*ldhA* carrying pTLP and pTLB. Fresh colonies were picked from an agar plate and inoculated into LB medium with chloramphenicol, the selection marker for pTLP and pTLB, and cultured at 37°C with shaking. This starter was counted as generation 0 with all bacteria bearing the plasmids. The cultures were then inoculated at a ratio of 1:1000 into fresh LB medium without antibiotics every 12 hours. Each subculture was considered 10 generations as bacteria growth reached 10 times the original cell numbers. After every 12 hours, bacteria were serially diluted and spread on an LB agar plate. The presence of the plasmids was determined by patching colonies onto two agar plates: one with antibiotics and one without. The fraction of plasmid-bearing bacteria was calculated as the percentage of antibiotic-resistant colonies over the total number of colonies. The experiment was conducted in biological triplicates, and no statistical differences were examined.

**Figure S2**


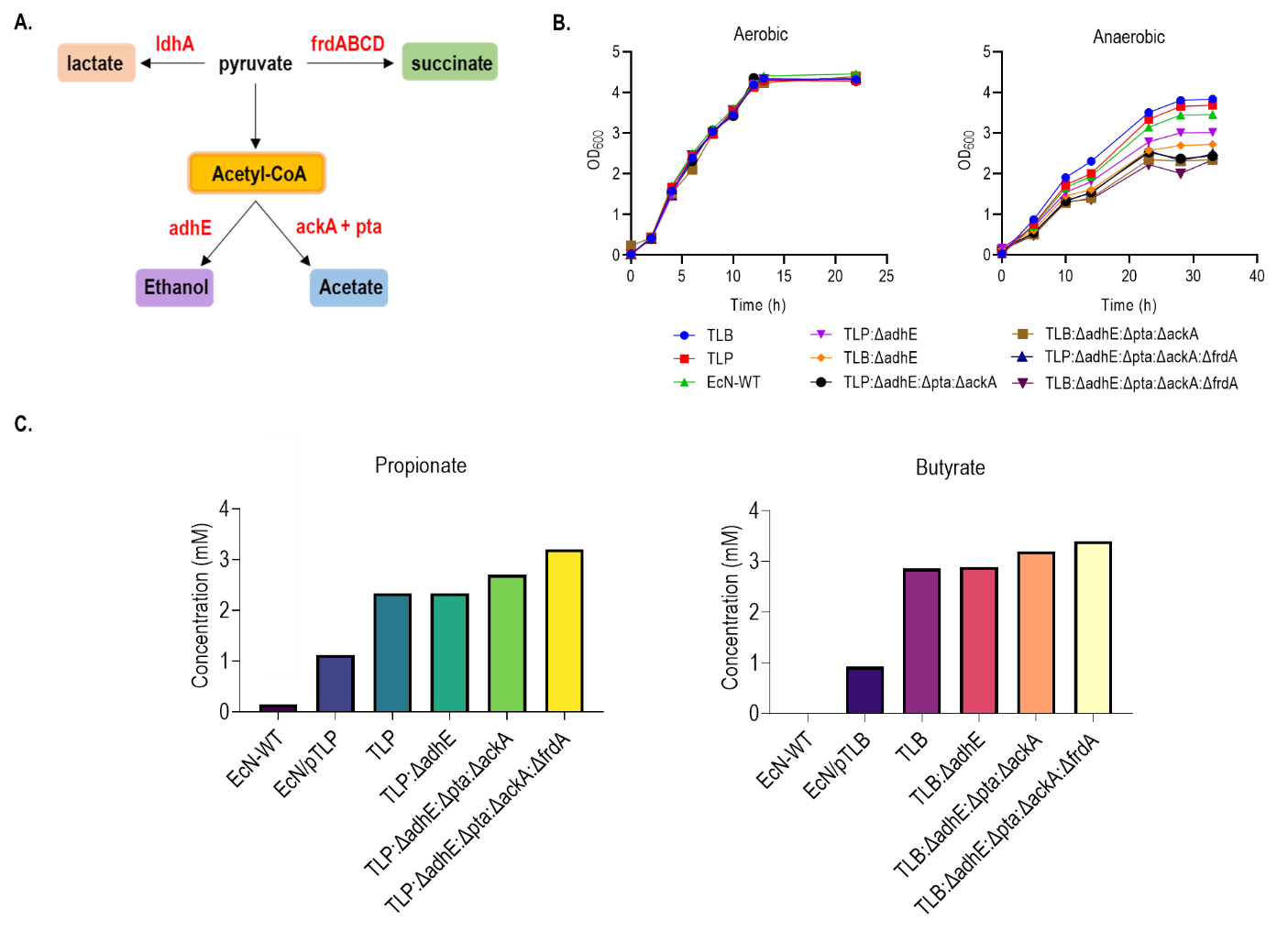


**Figure S2.** **Deletion of numerous competitive pathways decreases anaerobic growth. (A)** The competitive pathways of the synthetic propionate and butyrate pathways at the precursors: pyruvate and acetyl-CoA. ldhA: lactate dehydrogenase, frdABCD: fumarate reductase enzyme complex, adhE: aldehyde-alcohol dehydrogenase, ackA: Acetate kinase and pta: phosphate acetyltransferase. **(B)** The growth of wild-type EcN, the engineered strains TLP (EcN:Δ*ldhA*/pTLP) and TLB (EcN:Δ*ldhA*/pTLB) and other gene deletion strains in aerobic and anaerobic conditions using BHI medium. Bacteria from a single colony of each strain were cultured under the indicated conditions, and growth was measured by optical density (OD) at 600 nm (OD_600_). No statistical differences were examined. **(C)** Propionate and butyrate production of TLP and TLB were compared with other EcN variants of gene deletions carrying pTLP and pTLB. The measurements were performed on cultures from a single colony of each strain, and no statistical differences were examined.

**Figure S3**


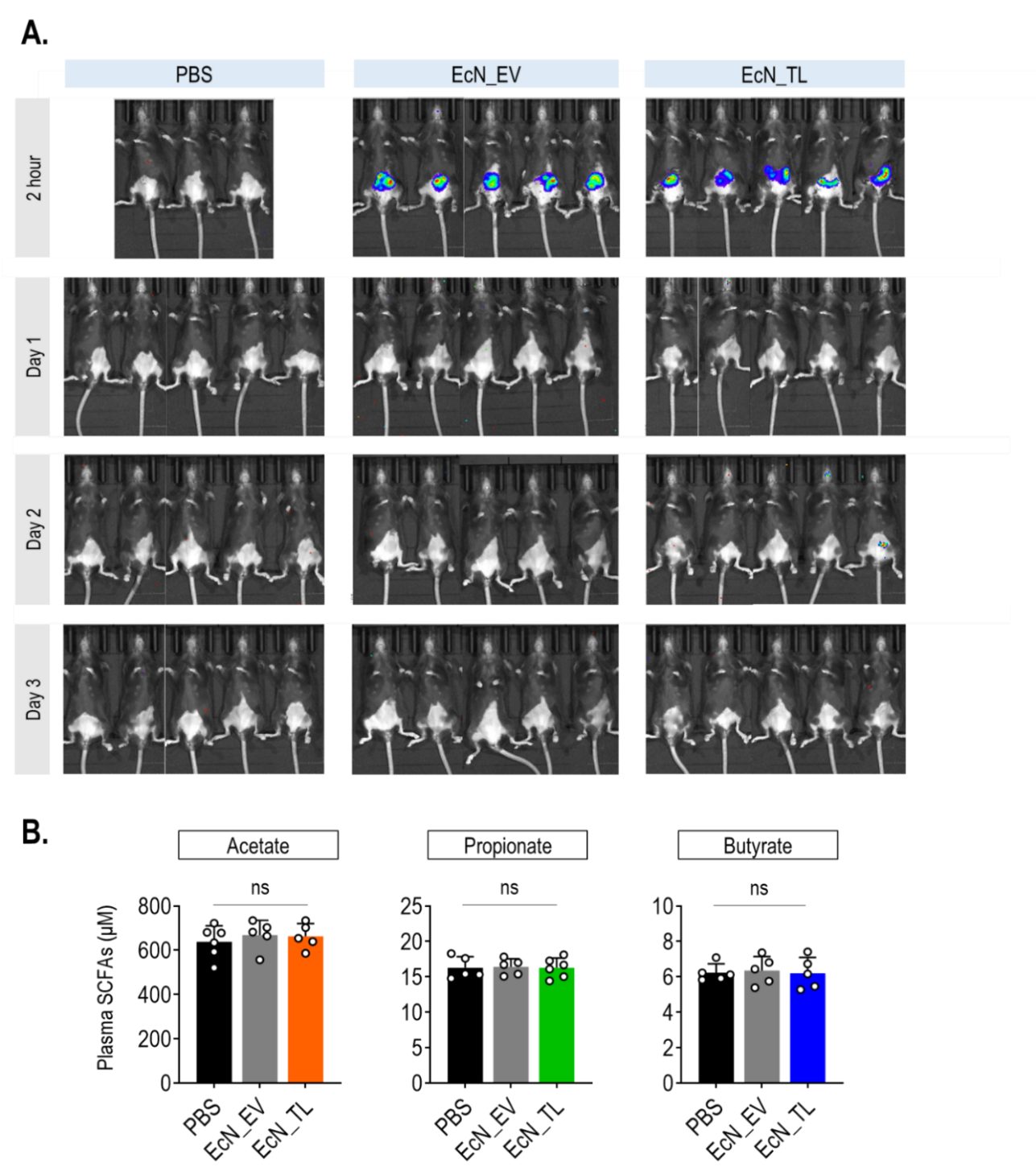


**Figure S3. Human-origin probiotics could not colonize and produce effect in the animal models without antibiotic treatment. (A)** Residence of bacteria in mouse gut visualized by luminescent intensity from the whole animal after feeding with Phosphate-buffered saline (PBS), EcN_EV (carrying empty vector), and engineered EcN_TL. After 14 daily doses of probiotics (10^9^ cfu/dose), the mice were sacrificed, and then the plasma was collected for SCFA measurements by GC-MS. **(B)** SCFA concentrations in plasma. Graphs represent changes in individual SCFAs (acetate, propionate, and butyrate). The data are represented as mean ± SEM. **p<0.01 compared with PBS control group; n = 5 biologically independent samples per group. One-way ANOVA was used for statistical analyses.

**Figure S4**


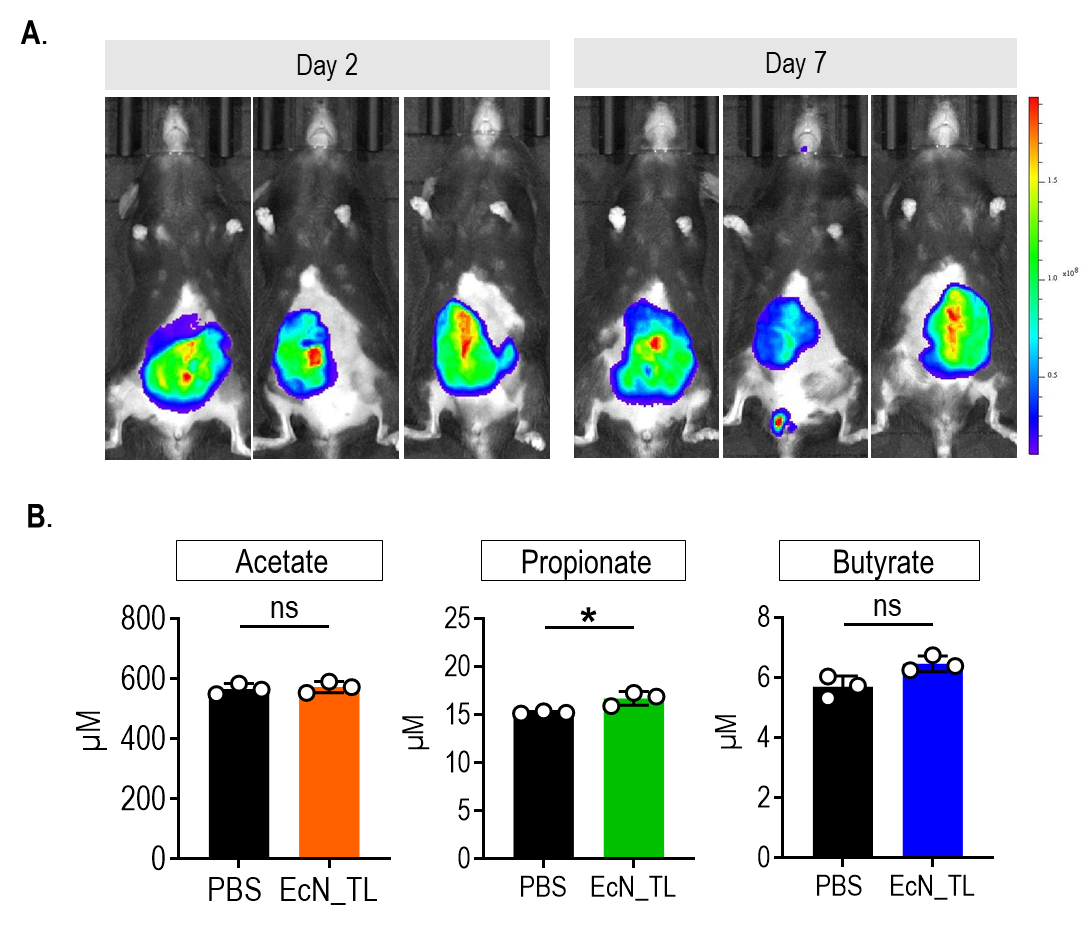


**Figure S4. The colonization and SCFA improvement in the blood of mice were daily fed with EcN_TL for 7 days. (A)** The residence of EcN_TL in mouse gut was visualized by luminescent intensity from the whole animal after antibiotic treatment followed by daily administration of EcN_TL. **(B)** Plasma SCFAs concentration on mice fed with 7 doses of EcN_TL. Graphs represent the changes in individual SCFAs (acetate, propionate, and butyrate). Statistical differences were examined by unpaired Student's t-test with n = 3. Symbols denote statistical significance, * = p<0.05.

**Figure S5**


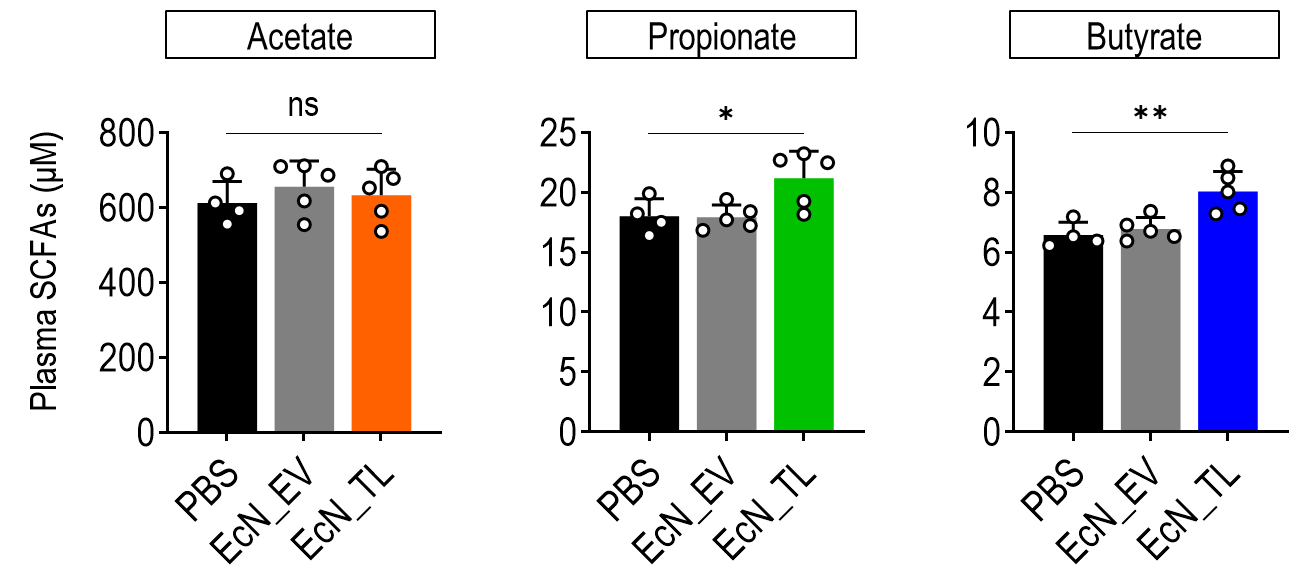


**Figure S5.** **SCFA concentrations in rat plasma**. Graphs represent changes in individual SCFAs (acetate, propionate, and butyrate). The data are represented as mean ± SEM. **p<0.01 compared with PBS control group; n = 5 biologically independent samples in EcN_EV and EcN_TL groups, n = 4 in PBS group. One-way ANOVA was used for statistical analyses.

**Figure S6**

**Figure S6**. **Echocardiography measurement of the individual rat**. The serial echocardiography measurement data of individual rat until 4 weeks after myocardial infarction. Left ventricular ejection fraction (EF), Left fractional shortening (FS), Left-ventricular internal diameter at end-diastole (LVIDd), Left-ventricular internal diameter at end-systole (LVIDs). n=7. The experiment was performed with seven animals for each group. No statistical differences were examined.

**Figure S7**

**Figure S7**. **Measurement of immune cells in blood after MI.** Immune cell type analysis in blood. Blood collecting was conducted from day 0 (D0) before myocardial infarction to day 14 (D14) after myocardial infarction. n=6. The experiment was performed with six animals for each group. Data are shown as the mean ± S.E.M. Statistical differences were examined by two-way ANOVA followed by Bonferrni’s post-hoc analysis. (*p<0.05 vs. Control).

**Figure S8**

**Figure S8.** SCFAs do not protect cardiac cells from ischemic stress. H9C2 myoblasts **(A)** and neonatal rat cardiomyocytes (NRCMs) **(B)** were incubated with individual SCFAs (50 μM of each SCFAs for H_9_C_2_ and 100 μM of each SCFAs for NRCM) or mixture on SCFAs (50 μM acetate, 50 μM propionate and 50 μM butyrate) for 18 hours followed with a treatment of hydrogen peroxide (H2O2) as indicated concentrations for 30 minutes. Cell survival was determined by the Cell Counting Kit-8 assay. Biological replicates, n = 3. Statistical differences were examined by one-way analysis of variance (one-way ANOVA). *p<0.05.

**Figure S9**

**
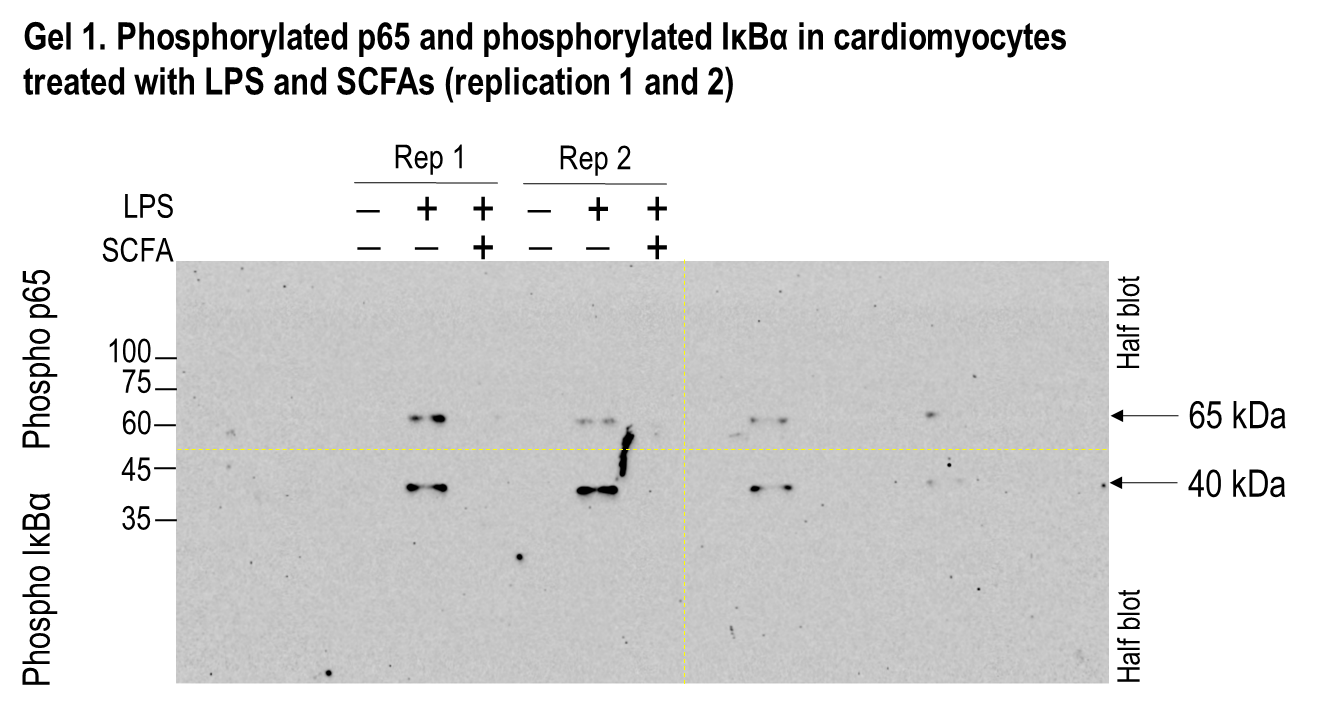
**

**
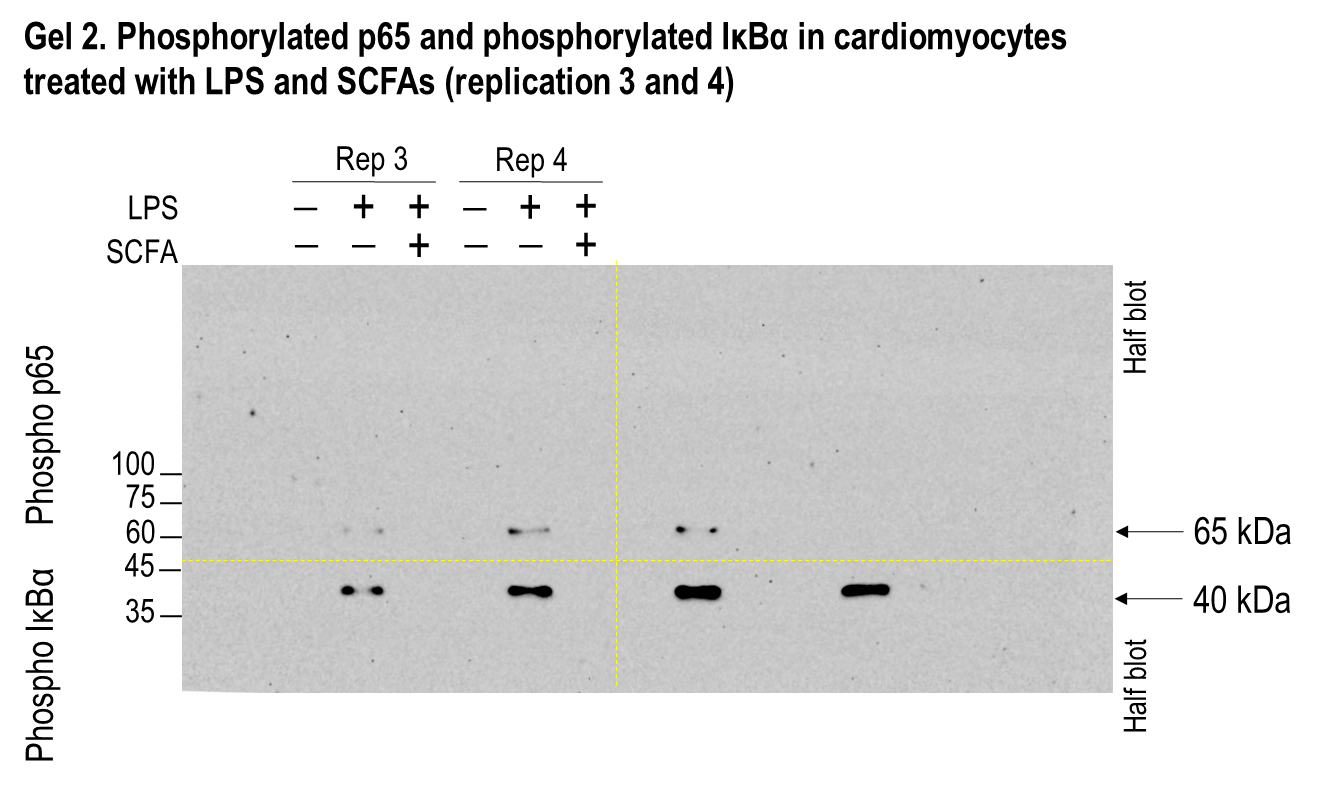
**

**
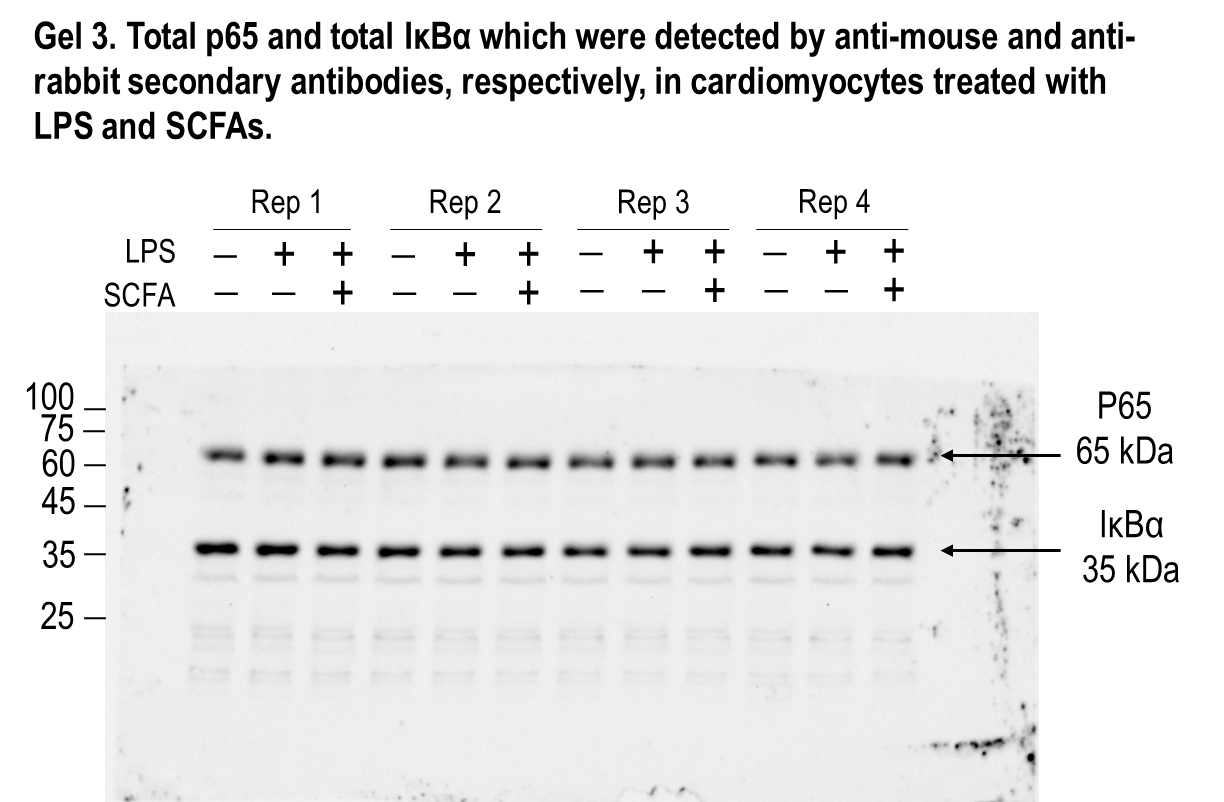
**

**
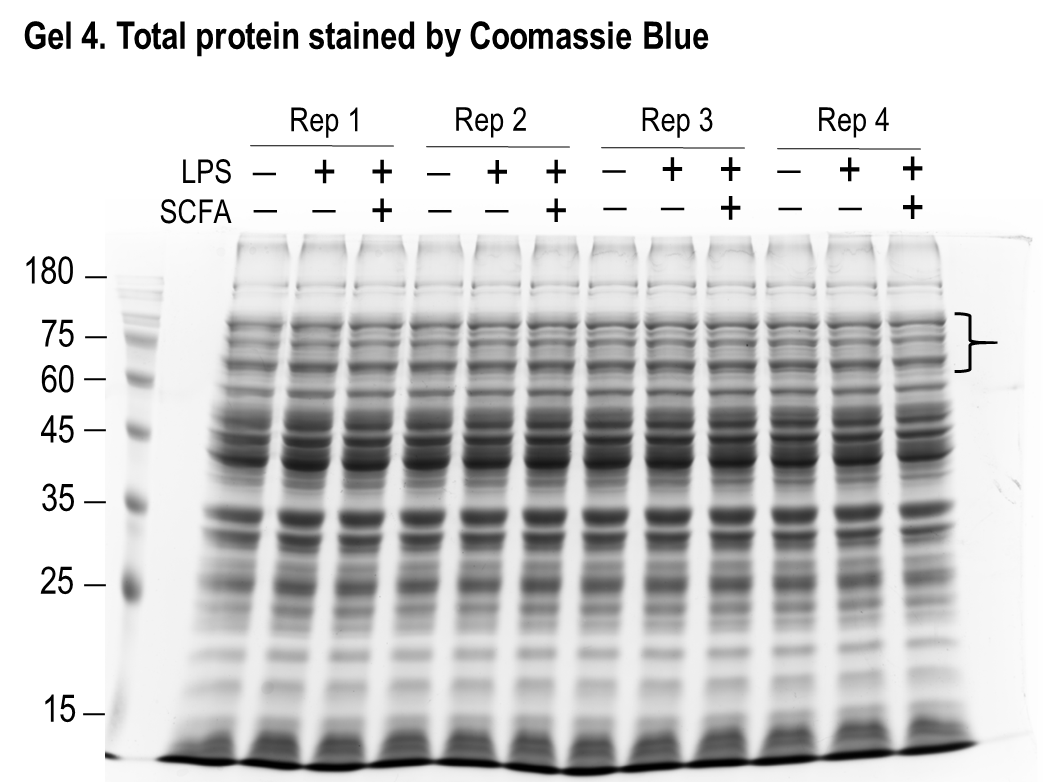
**

**Figure S9. Uncropped blot/gel images of Figure 6D.**

**Gel 1 and Gel 2: phosphorylated p65 and phosphorylated IκBα**. The membrane was cut in half and incubated separately with anti-NF-kB p65 phospho S536 and anti- IκBα phospho S36 antibodies. Both two primary antibodies are detected by anti-rabbit secondary antibody.

**Gel 3: total p65 and IκBα**. The whole membrane was incubated with anti-NF-kB p65 and anti- IκBα antibodies. Anti-NF-kB p65 and anti-IκBα antibodies were then detected by anti-mouse and anti-rabbit secondary antibodies, allowing the detection of two proteins on the same blot. **Gel 4: total protein stained by Coomassie Blue.**

**Figure S10**

**
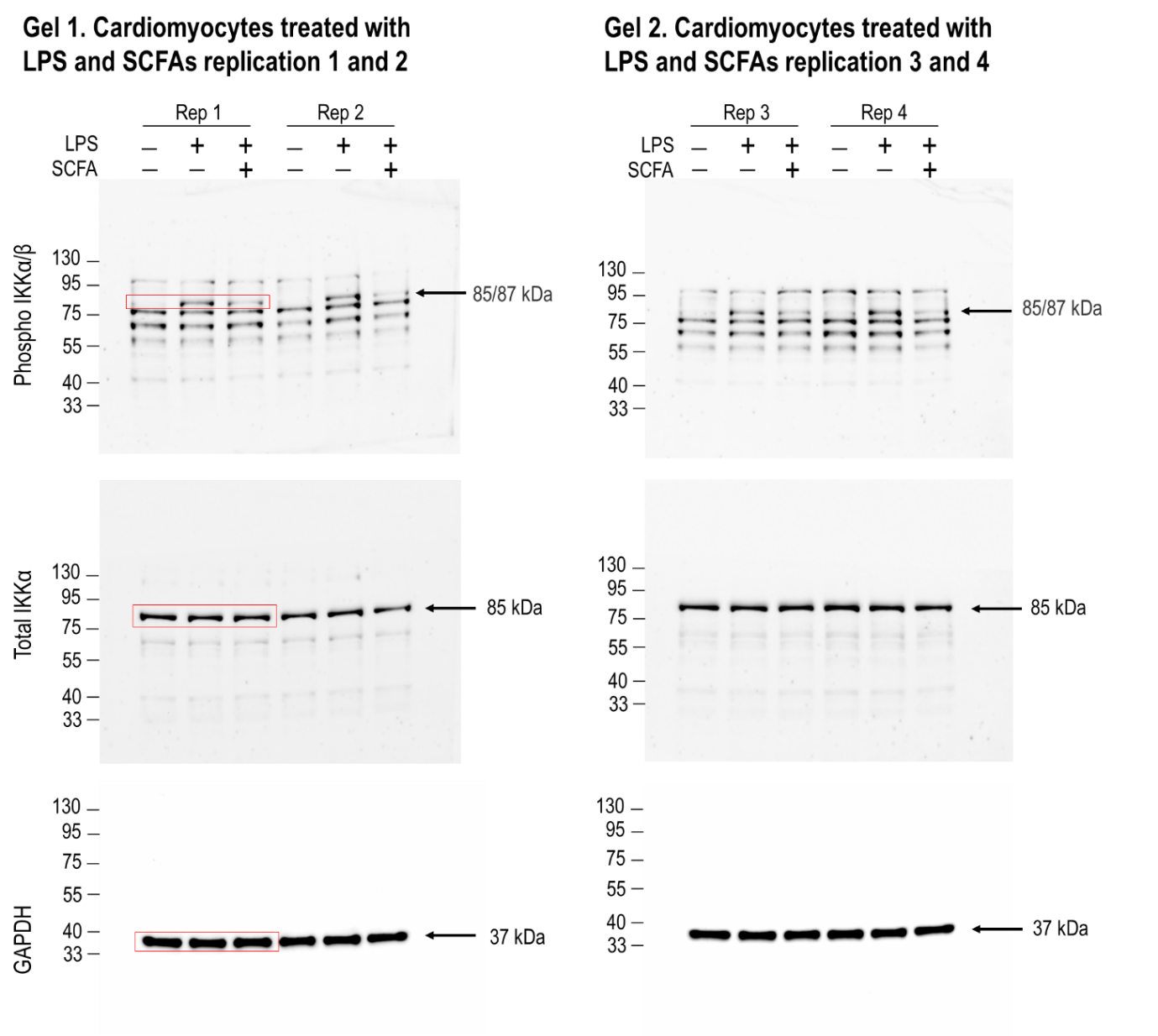
**

**Figure S10. Uncropped blot/gel images of Figure 6E.** Gel 1 consisted of replication 1 and 2; gel 2 consisted of replication 3 and 4. The detection of different proteins was performed on the same membrane by stripping the antibodies and incubating the membrane with another set. The order of detection for the primary antibodies is phosphorylated IKKα/β, total IKKα, and loading control GAPDH. The red boxes indicate the blots shown in figure 6E.

**Figure S11**

**
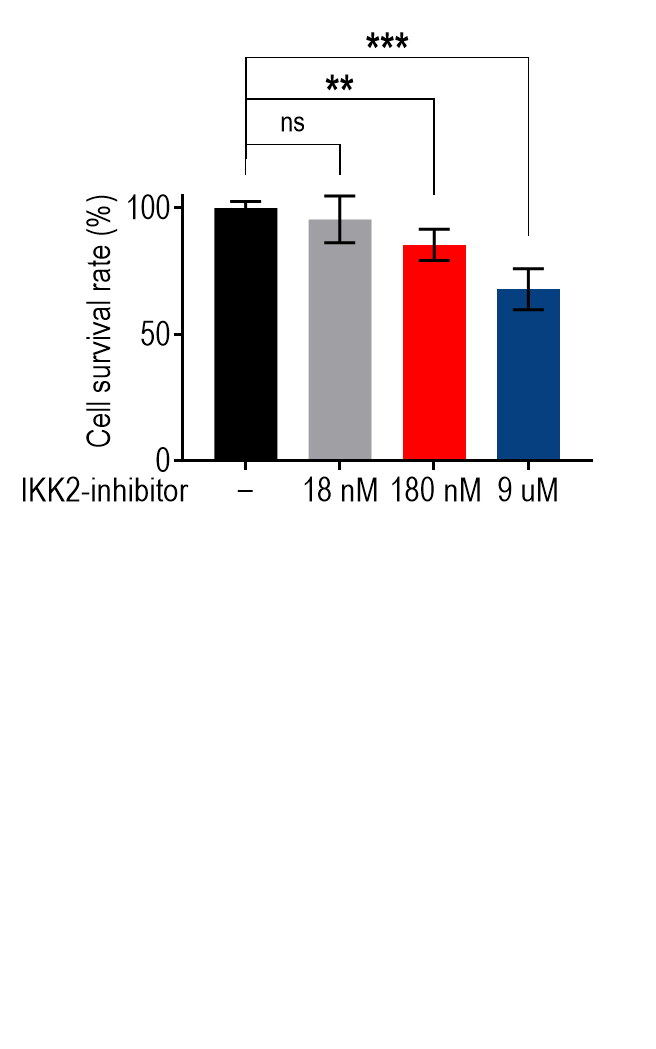
**

**Figure S11. Optimization of IKK2 inhibitor on cardiomyocyte survival.** IKK2 at 18nM did not significantly affect the survival of the cells. Therefore, the concentration of 18nM was chosen for other experiments in this study regarding the impacts on SCFA on NF-kB pathway.

**Figure S12**

**
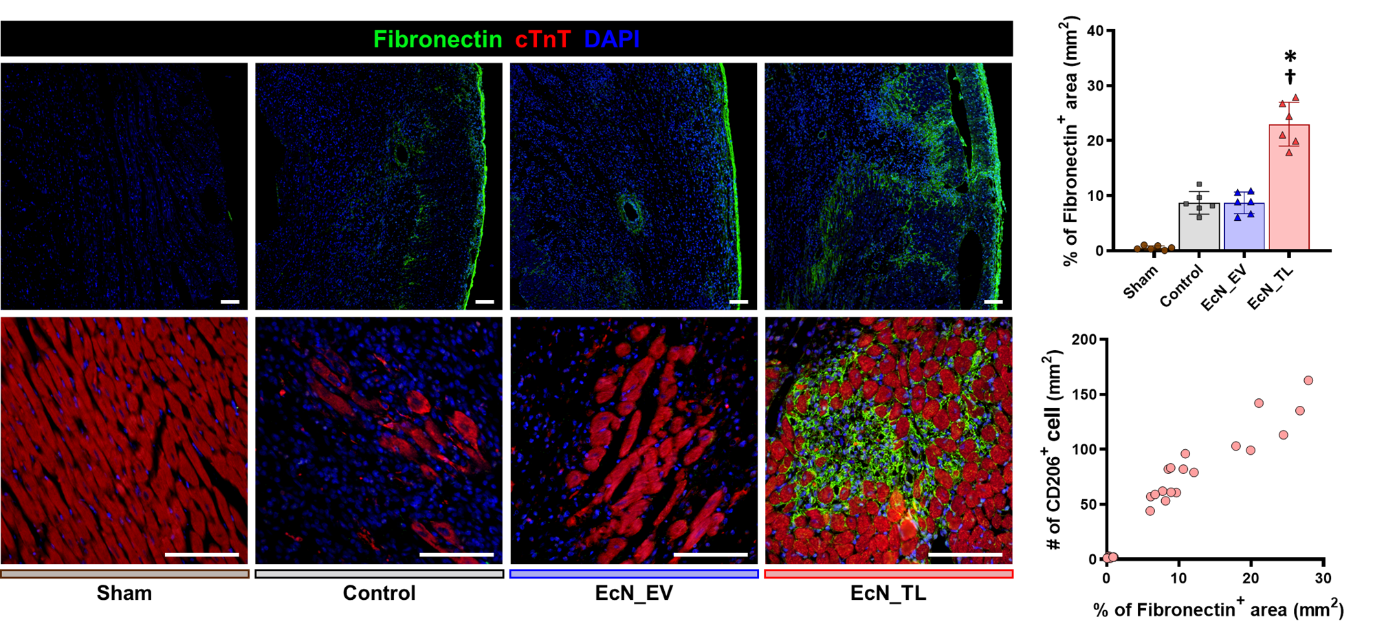
**


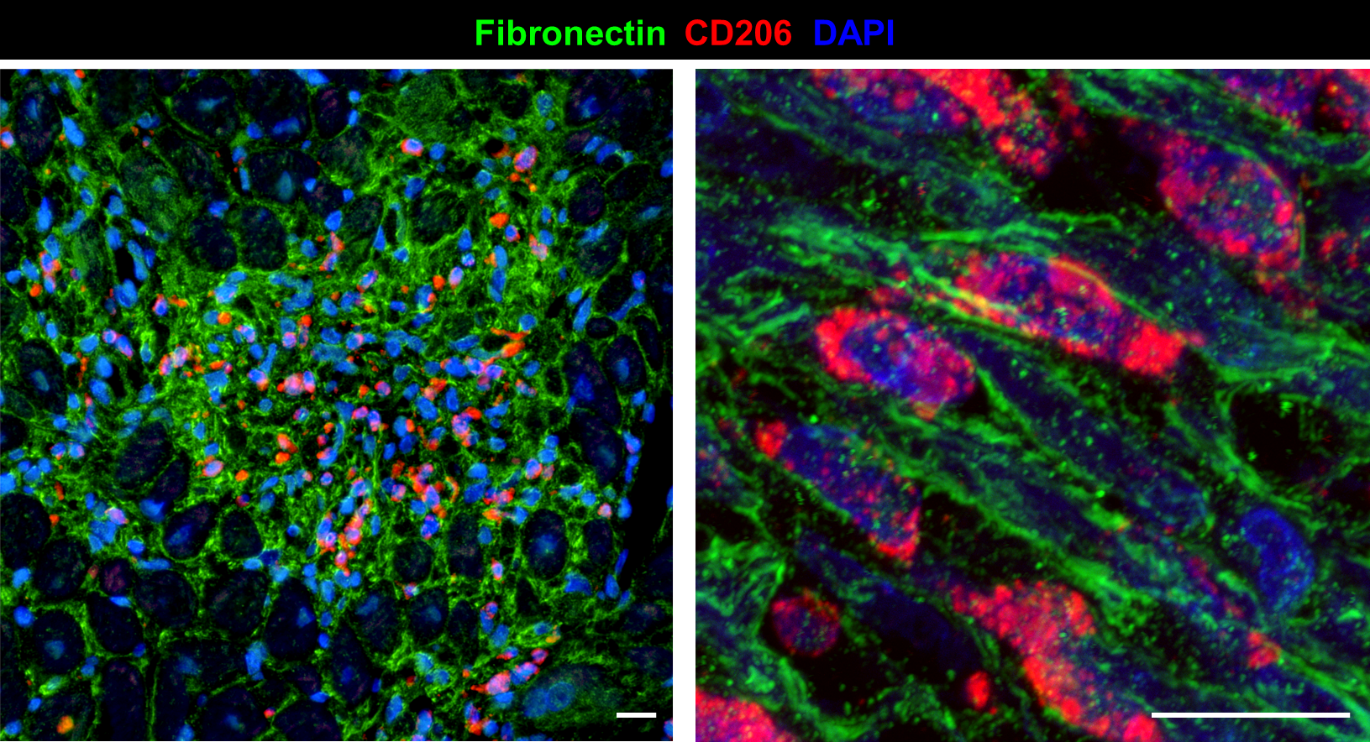


**Figure S12. Representative images of fibronectin staining at 3 days after myocardial infarction and the quantification summary**. Fibronectin (Green), cTnT or CD206 (Red), DAPI (Blue). n=6. Scale bar: 200 µm (upper panel) and 5 µm (lower panel). Data are shown as the mean ± S.E.M. Statistical differences between the three groups were examined by one-way ANOVA followed by Bonferroni's post-hoc analysis. (*p<0.05 vs. Control. †p<0.05 vs. EcN_EV).

**Figure S13**

**
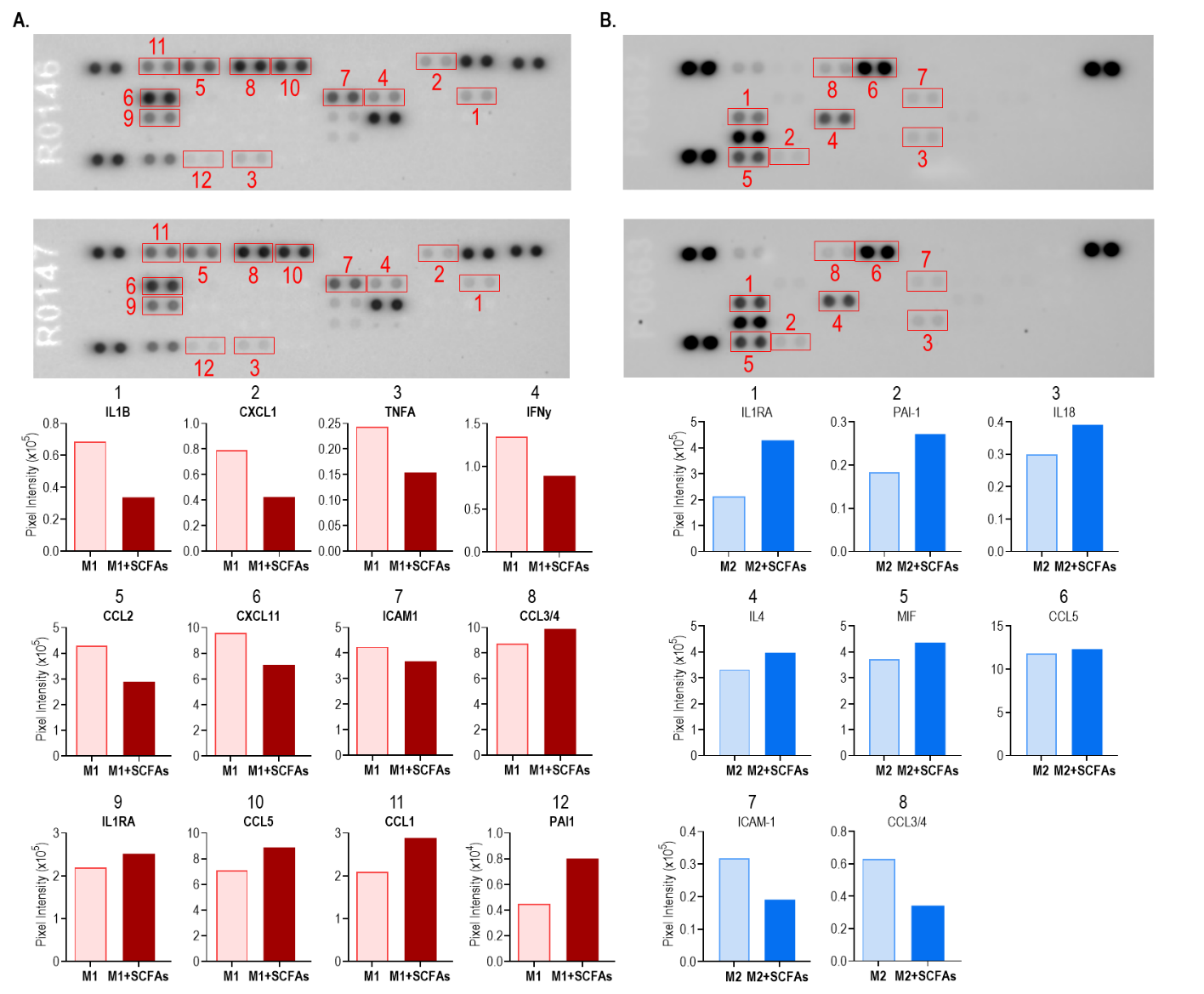
**

**Figure S13. (A-B)** SCFAs upregulate anti-inflammatory cytokines and suppress pro-inflammatory cytokines respectively in macrophages under both M1 and M2 conditions. Cytokine array results for macrophages under M1 **(A)** and M2 **(B)** conditions in the presence and absence of SCFAs. Biological replicates, n =1. No statistical differences were examined.

**Figure S14**

**
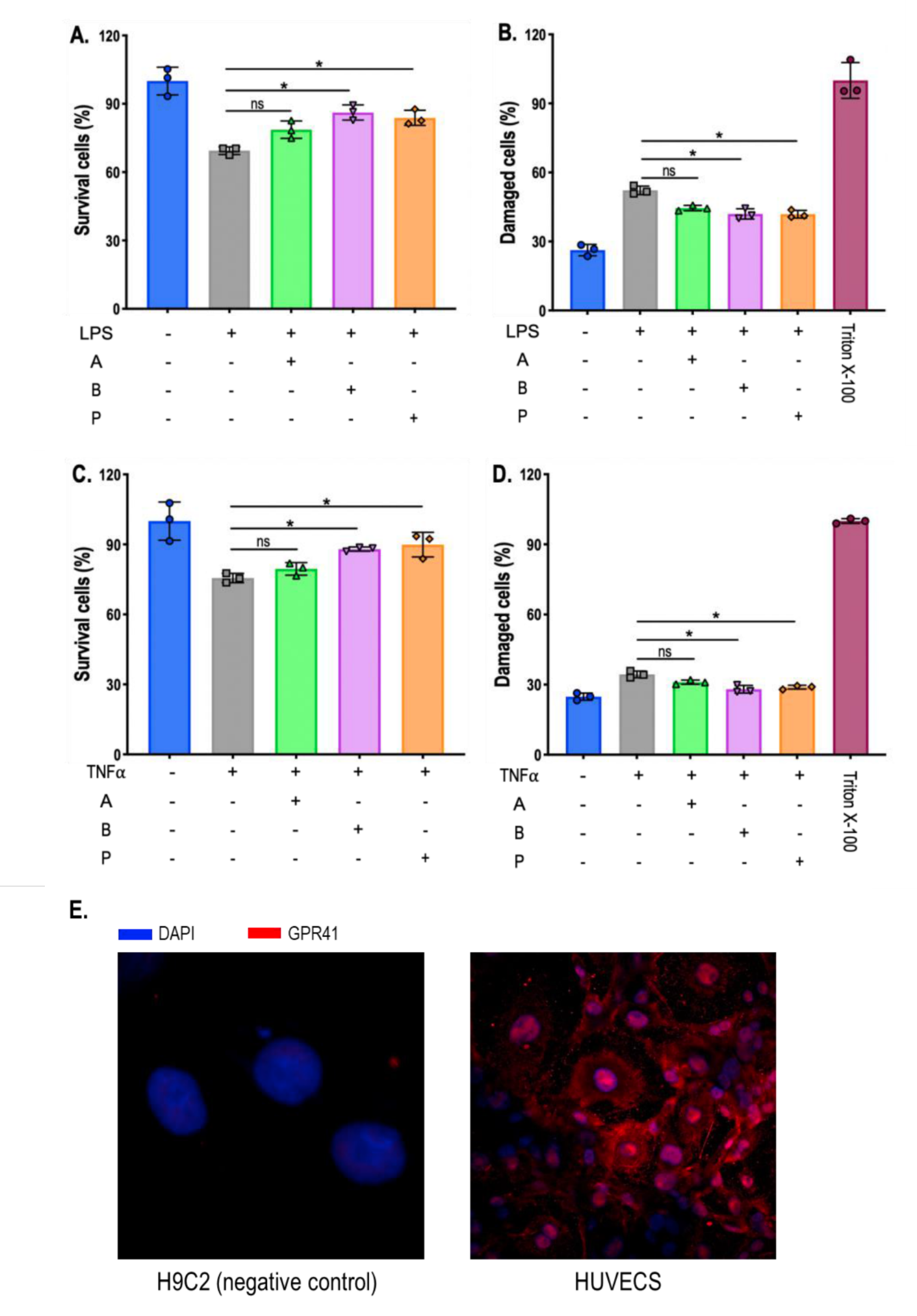
**

**Figure S14.** SCFAs protected endothelial cells from inflammatory injury**. (A-D)** SCFAs improve cell survival during inflammation in endothelial cells. The SCFA treatment was similar to cardiomyocytes followed by LPS **(A, B)** or TNF-α **(C, D)** stimulation. Cell survival was measured by CCK8 assay, and cell damage was determined by LDH assay. Biological replicates, n = 3. Statistical differences were examined by one-way ANOVA. *p <0.05. (E) Immunocytochemistry image of GPR41 expressed in endothelial cell HUVEC.

**Figure S15**

**
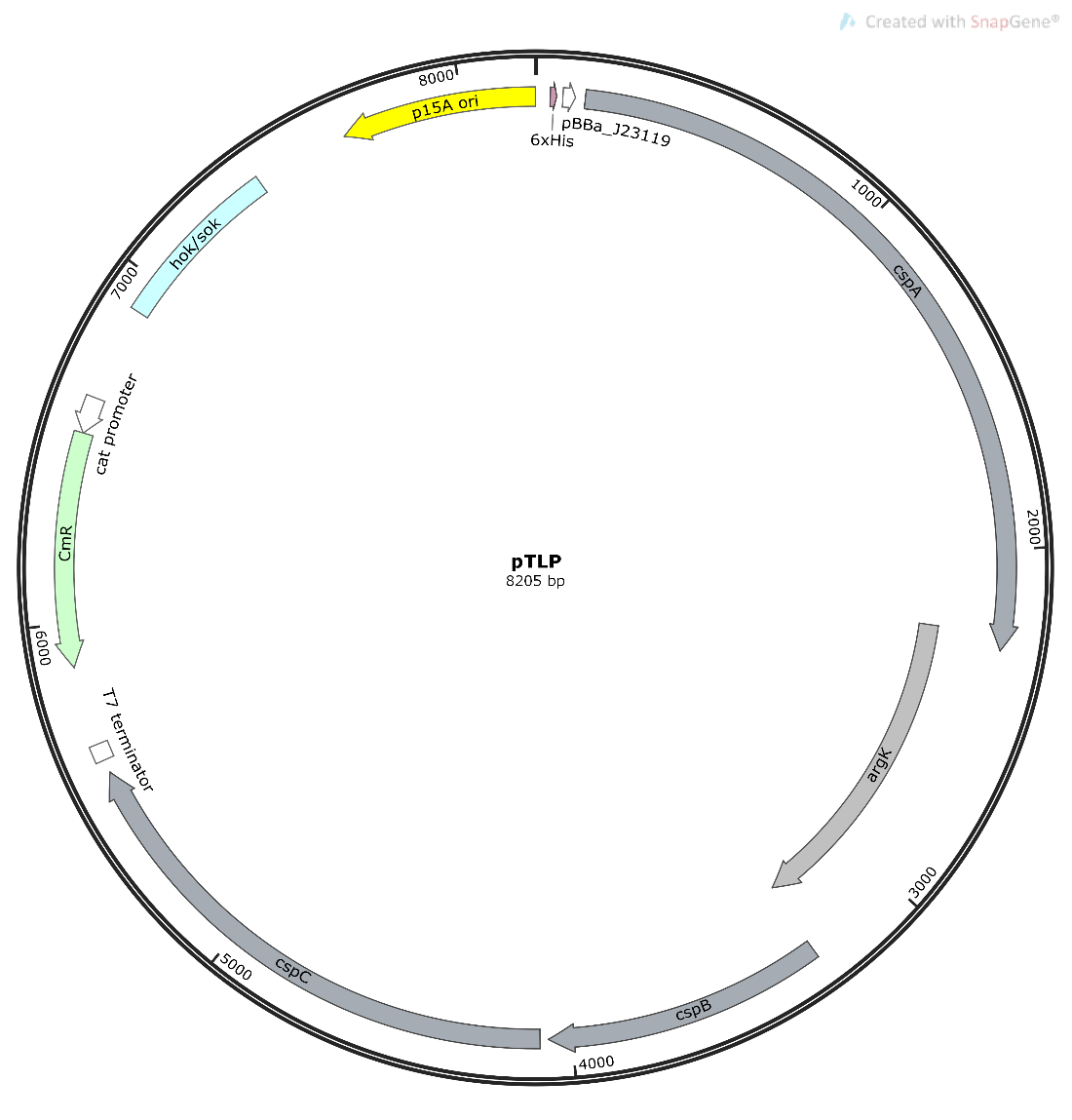
**

**Figure S15. The map sequence of the plasmid pTLP**

**Figure S16**

**
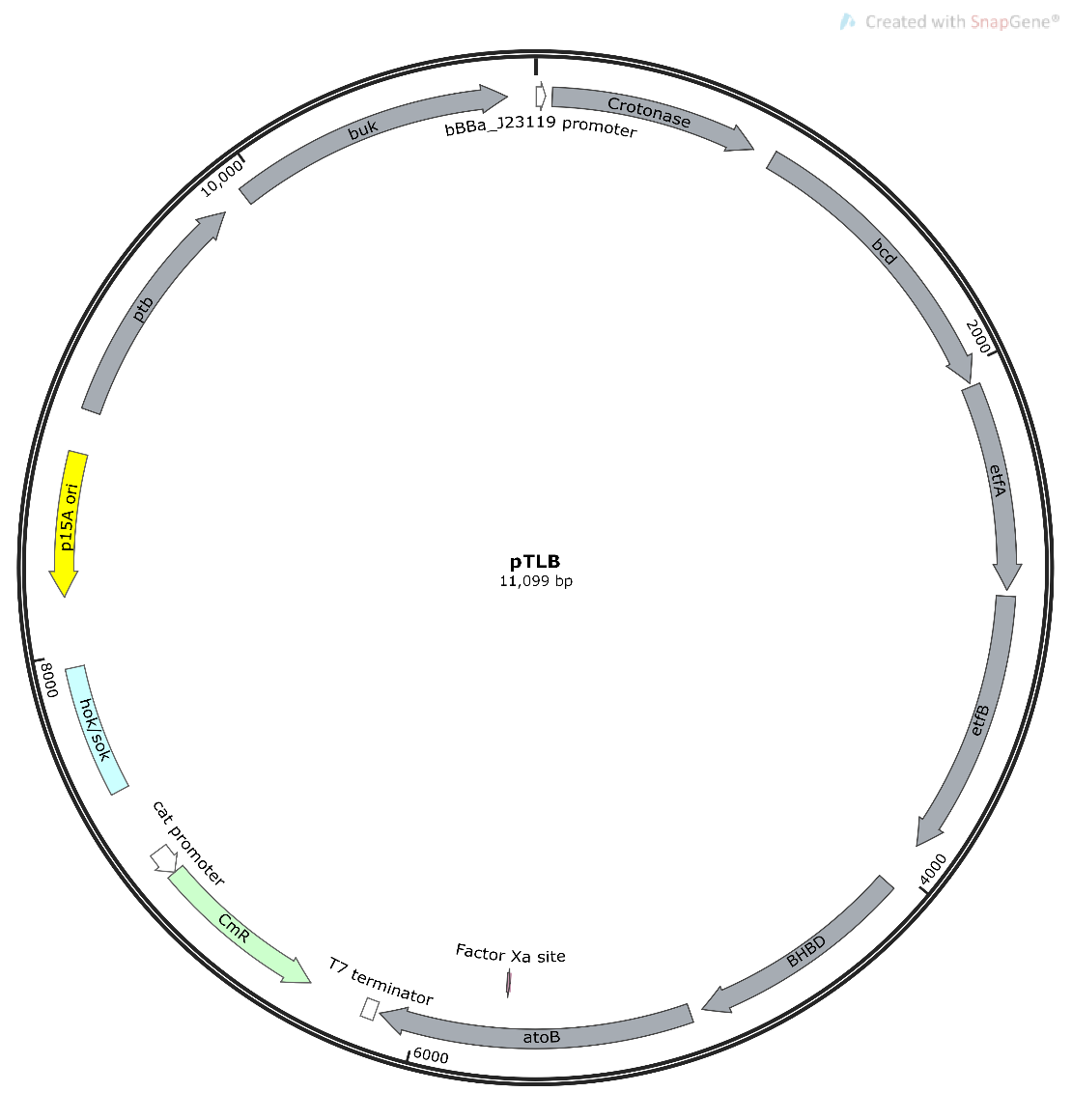
**

**Figure S16. The map sequence of the plasmid pTLB**

**Table S1. List of oligonucleotides used in the study.**

| Name | SEQUENCE (5’-3’) | Source |
| --- | --- | --- |
| hok/sok F | ATCGGCTAGCTGATGCGGCAACAATCACAC | This study |
| hok/sok R | TGCA GCTAGC AGTCAGACCAGCATCAGTCC | This study |
| sbm F | TAGAGTTTAAGGAGATATACATATGTCTAACGTGCAGGAGTGGCAAC | This study |
| sbm R | CAGCGGTGGCAGCAGCCTAGGTTAATTAACCCAGCATCGAGCCGGTTG | This study |
| atoB F | AATATCCCGTTAAATAAATATAGGAGGTTAAGTAATGAAAAATTGTGTCATCGTC | This study |
| atoB R | GCTAGTTATTGCTCAGCGGTTTAATTCAATCGTTCAATCACC | This study |
| BHBD F | GCTTCTAGGAGTATATTTATTTAAC | This study |
| BHBD R | ACCGCTGAGCAATAAC | This study |
| ptb-delete F | ATTAATCAGATAAAATATTTATTTTCAGAAAATTTAGCATTTAAAG | This study |
| ptb-delete R | ACTGAGCTAGCTGTAAAGAACATTTTTATAAATTCCATTTTTTCC | This study |
| ldhA-delete F | ATGAAACTCGCCGTTTATAGCACAAAACAGTACGACAAGAAGTACCTGCAAATATCCTCCTTAGTTCCTATTCCG | This study |
| ldhA-delete R | TTAAACCAGTTCGTTCGGGCAGGTTTCGCCTTTTTCCAGATTGCTTAAGTCTGCTTCGAAGTTCCTATACTTTC | This study |
| adhE-delete F | ATGGCTGTTACTAATGTCGCTGAACTTAACGCACTCGTAGAGCGTGTAAAAATATCCTCCTTAGTTCCTATTCCG | This study |
| adhE-delete R | TTAAGCGGATTTTTTCGCTTTTTTCTCAGCTTTAGCCGGAGCAGCTTCTTCTGCTTCGAAGTTCCTATACTTTC | This study |
| pckA-delete F | TGTCAAATATGAATTTCTCCAGATACGTAAATCTATGAGC GAACTTCAGAGCGCTTTTG | This study |
| pckA-delete R | AATATGTATTGCCTGAATAGTAAAGTCTTTTTGGGGGTGT GGTCACAGCTTGTCTGTAAG | This study |
| frdA-delete F | TAAAAAAAGCACGATCTGATGGTTTAGTAATTAAATTAATCATCTTCAGTAATATCCTCCTTAGTTCCTATTCCG | This study |
| frdA-delete R | GTTGCGTCATAAGGCACTTCATAGAATGCGCTATGCGGTGCGGTATCGACCTGCTTCGAAGTTCCTATACTTTC | This study |
| pta-delete F | TGTAACCCGCCAAATCGGCGGTAACGAAAGAGGATAAACCGTGTCCCGTATTATTATGCTG | This study |
| pta-delete R | TTCAGATATCCGCAGCGCAAAGCTGCGGATGATGACGAGATTACTGCTGCTGTGCAG | This study |
| r-IL 1b F | AGAAGAGCCCGTCCTCTGTGA | Lab collection |
| r-IL 1b R | TCAGACAGCACGAGGCATTT | Lab collection |
| r-TNF F | GCATGATCCGAGATGTGGAA | Lab collection |
| r-TNF R | CAGACACCGCCTGGAGTTCT | Lab collection |
| r-IL10 F | GAATTCCCTGGGAGAGAAGC | Lab collection |
| r-IL10 R | CGGGTGGTTCAATTTTTCAT | Lab collection |
| r-IL6 F | CACTTCACAAGTCGGAGGCT | This study |
| r-IL6 R | TCTGACAGTGCATCATCGCT | This study |
| h-TNFα F | TCCCCAGGGACCTCTCTCTA | Lab collection |
| h-TNFα R | GGGTTTGCTACAACATGGGCTA | Lab collection |
| h-IL1β F | ATGATGGCTTATTACAGTGGCAA | Primerbank (primer pair 1) |
| h-IL1β R | GTCGGAGATTCGTAGCTGGA |  |
| h-IL1RA F | GAAGATGTGCCTGTCCTGTGT | ^1^ |
| h-IL1RA R | CGCTCAGGTCAGTGATGTTAA |  |
| h-FN F | GAGAATAAGCTGTACCATCGCAA | Primerbank (primer pair 3) |
| h-FN R | CGACCACATAGGAAGTCCCAG |  |

1. Kalliolias, G.D., Gordon, R.A. & Ivashkiv, L.B. Suppression of TNF-alpha and IL-1 signaling identifies a mechanism of homeostatic regulation of macrophages by IL-27. *J Immunol* **185**, 7047-56 (2010).
